# Equine maternal aging affects the metabolomic profile of oocytes and follicular cells during different maturation time points

**DOI:** 10.3389/fcell.2023.1239154

**Published:** 2023-09-25

**Authors:** G. D. Catandi, D. R. Bresnahan, S. O. Peters, K. J. Fresa, L. J. Maclellan, C. D. Broeckling, E. M. Carnevale

**Affiliations:** ^1^ Department of Biomedical Sciences, College of Veterinary Medicine and Biomedical Sciences, Colorado State University, Fort Collins, CO, United States; ^2^ Department of Animal Sciences, Berry College, Mount Berry, GA, United States; ^3^ Proteomic and Metabolomics Core Facility, Colorado State University, Fort Collins, CO, United States

**Keywords:** mare, oocyte, age, maturation, cumulus, granulosa, follicle, metabolome

## Abstract

**Introduction:** Oocyte quality and fertility decline with advanced maternal age. During maturation within the ovarian follicle, the oocyte relies on the associated somatic cells, specifically cumulus and granulosa cells, to acquire essential components for developmental capacity.

**Methods:** A nontargeted metabolomics approach was used to investigate the effects of mare age on different cell types within the dominant, follicular-phase follicle at three time points during maturation. Metabolomic analyses from single oocytes and associated cumulus and granulosa cells allowed correlations of metabolite abundance among cell types.

**Results and Discussion:** Overall, many of the age-related changes in metabolite abundance point to Impaired mitochondrial metabolic function and oxidative stress in oocytes and follicular cells. Supporting findings include a higher abundance of glutamic acid and triglycerides and lower abundance of ceramides in oocytes and somatic follicular cells from old than young mares. Lower abundance of alanine in all follicular cell types from old mares, suggests limited anaerobic energy metabolism. The results also indicate impaired transfer of carbohydrate and free fatty acid substrates from cumulus cells to the oocytes of old mares, potentially related to disruption of transzonal projections between the cell types. The identification of age-associated alterations in the abundance of specific metabolites and their correlations among cells contribute to our understanding of follicular dysfunction with maternal aging.

## Introduction

Maternal aging is associated with a reduction in oocyte quality and a decline in fertility in mammalian species. This is most widely documented in women, with a notable decline in oocyte quality and fertility after women are approximately 35 years of age (Krisher, 2019a). The mare is a monovular, large-animal species with similarities to women in follicular dynamics and reproductive aging (Carnevale, 2008; Carnevale et al., 2020). In general, fertility declines as mares enter their teen years, with a more precipitous decline after 20 years of age (McDowell et al., 1992). Transfers of oocytes from young and old mares into the oviducts of young, inseminated recipient mares demonstrated that a decline in oocyte quality was a significant cause of the age-associated reduction in fertility (Carnevale and Ginther, 1995). When compared to samples from young mares, oocytes and 2-day old embryos from old mares have significantly reduced metabolic capacity and less potential to develop into blastocysts after fertilization by intracytoplasmic sperm injection (Catandi et al., 2021). The metabolome of oocytes and the surrounding follicular cells of young and old mares at different maturation stages has not been previously compared. However, elucidating specific changes in the ovarian follicle promoted by maternal aging could aid in our understanding of the age-associated decline in fertility and the identification of biomarkers and therapeutic approaches.

Oocyte meiotic maturation is the final stage of oocyte development that culminates, in most mammalian species, with ovulation of a developmentally competent oocyte. Oocyte maturation naturally occurs within the ovarian follicle and is tightly regulated by hormonal signals and bidirectional communications between the oocyte and follicular cells (Sugiura et al., 2005). Granulosa cells line the ovarian follicle and participate in steroidogenesis; they are essential for the transport of nutrients from the systemic circulation into the follicular fluid, creating a local environment for the developing oocyte (Siu and Cheng, 2013). Cumulus cells are a specialized type of granulosa cells that directly surround and contact the oocyte through transzonal projections; these cellular projections transverse the zona pellucida and allow cumulus cells to transfer compounds, including signaling molecules and energy substrates, via gap junctions to the oocyte (Richani et al., 2021). Therefore, the oocyte is dependent on the follicular cells to provide essential components to maintain its developmental capacity and coordinate oocyte and follicular maturation.

The oocyte undergoes nuclear and cytoplasmic changes during maturation, which help it acquire the ability to properly undergo fertilization and embryo development. Cytoplasmic maturation includes mitochondrial replication, cortical granule and organelle re-organization, and accumulation of maternal RNA and proteins, with the consumption of energy substrates critical to support these processes (Warzych and Lipinska, 2020; Strączyńska et al., 2022). Gap junctions and transzonal projections from cumulus cells to the oocyte are disrupted during the final stages of oocyte maturation (Turathum et al., 2021). However, until the completion of maturation, direct contact between cumulus cells and the oocyte is important to maximize the transfer of nutrients to the energy-demanding oocyte. Disruptions of follicular-cell communications may be associated with maternal aging, resulting in altered oocyte maturation and developmental competency. In female mice, aging reduces the abundance of transzonal projections (El-Hayek et al., 2018), which could limit the transfer of molecules from cumulus cells to oocytes. Consequently, oocytes from older animals may not obtain adequate nutrients to fuel the energy-dependent mechanisms associated with maturation, fertilization, and subsequent cell divisions during early embryo development.

In the mare as in the woman, the dominant follicle has a long follicular phase with approximately 1.5 days from maturation induction to ovulation (Gérard and Robin, 2019). Equine oocytes collected at approximately 1 day after induction of follicle maturation will continue to mature in hormone-free medium (Carnevale, 2016), with high maturation rates and developmental potential (Frank et al., 2019). However, the extent that exposure of the maturing oocyte or follicle cells to culture medium results in alterations of the metabolome is not known. In the present study for the final stage of maturation, we exposed maturing oocytes and follicle cells to a culture system and medium that has been used to consistently produce pregnancies and offspring in a clinical program (Carnevale, 2016; Frank et al., 2019).

In the present study, we obtained the metabolomic profile of oocytes, cumulus cells, and granulosa cells during different stages of maturation of dominant, follicular-phase follicles from young and old mares. Our first aim was to determine age-associated differences in the metabolomes of oocytes and follicular cells during three approximate stages of equine oocyte maturation (immature/germinal vesicle; maturing/metaphase I; and mature/metaphase II), and our second aim was to determine how metabolite abundance was correlated among follicular cell types (oocytes, cumulus cells and granulosa cells). Our findings contribute to the understanding of how maternal aging affects the follicular microenvironment and provides potential biomarkers of oocyte quality and a basis for therapeutic interventions.

## Materials and methods

### Study design

Metabolomic assessments were performed for oocytes, cumulus cells (CC) and granulosa cells (GC) collected from individual, follicular-phase, dominant follicles and compared between young mares and old mares at three stages of follicle/oocyte development: 1) 0 h, prior to follicular maturation at the germinal vesicle stage of oocyte development, 2) 24 h, approximately 24 h after follicle maturation induction and at the anticipated metaphase I stage of oocyte development, and 3) 42 h, with cell collections from mares at 24 h after follicle maturation induction and followed by 18 h of culture, with oocytes confirmed at the metaphase II stage of development.

### Animals

Procedures performed in this study were approved by the Colorado State University’s Institutional Animal Care and Use Committee. Follicular cell samples were collected between June and August, from young mares (Young, 7–11 years, mean age of 10 ± 0.6 years, *n* = 8) and old mares (Old, 19–28 years, mean age of 23 ± 0.8 years, *n* = 12). Mares were housed in dry lot paddocks with access to covered shelters and fed a mixture of grass and alfalfa hay at approximately 2% body weight daily; mineral salt and water were provided *ad libitum*.

### Sample collections from preovulatory follicles

Mares’ reproductive tracts were examined by transrectal ultrasonography, using a 7.5 MHz linear probe, to monitor ovarian activity. Granulosa cells and cumulus oocyte complexes were collected by transvaginal, ultrasound-guided, follicular aspiration of dominant follicles, as described (Carnevale, 2016). For time 0 (0 h), samples were collected from follicles ≥ 35 mm in diameter when endometrial edema indicative of estrus was observed and without induction of follicular maturation. Cumulus and granulosa cells were assessed to assure compact cumulus and granulosa cells that were consistent with an immature oocyte at the germinal vesicle stage. For 24 and 42 h samples, follicular maturation was induced by administration of human chorionic gonadotropin (2,000 IU, intravenous; Chorulon, Merck Animal Health, Madison, NJ) and deslorelin acetate in an aqueous base (0.75 mg, intramuscular; Precision Pharmacy, Bakersfield, CA) when follicles and endometrial edema were consistent with 0 h collections. Follicular samples were collected at 24 ± 2 h after induction for the 24 and 42 h groups, and samples were determined to be consistent with a maturing follicle, with expanding cumulus and granulosa cells. For 42 h samples, cumulus oocyte complexes and sheets of granulosa cells were incubated separately in tissue culture media 199 with Earle’s salts (GibcoTM, Thermo Fisher, Waltham, MA) with additions of 10% fetal calf serum, 25 μg/mL of gentamicin, and 0.2 mM pyruvate at 38.2°C in an atmosphere of 5% CO_2_ and air for 18 ± 2 for completion of maturation. Oocyte maturation was considered complete (metaphase II) at approximately 42 h after the administration of induction drugs to mares, which is the timeline used in the laboratory for fertilization of equine oocytes from dominant follicles (Frank et al., 2019). At 0, 24 or 42 h, oocytes were denuded of cumulus cells by sequential pipetting in a MOPS-buffered medium (G-MOPS™, Vitrolife, Englewood, CO) with 0.04% bovine serum albumin (BSA; Sigma-Aldrich, St Louis, MO) and hyaluronidase (200 IU/mL; Sigma-Aldrich), and oocytes were evaluated under magnification to confirm the complete removal of cumulus cells. Extrusion of the first polar body was confirmed for oocytes at 42 h to establish the metaphase II stage of development. Oocytes, cumulus cells, and granulosa cells from individual follicles were rinsed and fixed separately in 20 μL (granulosa and cumulus cells) or 10 μL (oocytes) of 50% methanol solution and stored in glass vials at −80°C until analyses.

### Oocyte, cumulus and granulosa cell metabolite extraction and detection using liquid and gas chromatography coupled to mass spectrometry

Samples were frozen to −80°C and thawed to 4°C three times before the addition of 250 μL of 100% methanol. Samples were then sonicated in a QSonica ultrasonic processor at 65% amplitude for 10 min, before being vortexed for 2 h at 4°C. After centrifugation at 3000 x g at 4°C, two individual aliquots of 120 μL of extract were transferred into 2-mL glass vials and dried under nitrogen gas for mass spectrometry analyses by liquid chromatography (LC-MS) and gas chromatography (GC-MS). The number of granulosa and cumulus cells per sample was not known, so the remaining sample pellet was used to estimate biomass through quantification of protein by reconstitution with urea and measurement of absorbance at 280 nm using a NanoDrop™ spectrophotometer (Thermo Fisher). Two distinct drops were measured in triplicate for each sample; the highest and lowest readings were discarded, and the remaining values were averaged.

For LC-MS analysis, granulosa and cumulus cell extracts were resuspended in volumes proportional to their protein content (5 μL of 100% methanol was used per 5 μg/μL of protein content) with a minimum volume of 25 μL. Oocyte samples had a protein concentration less than 5 μg/μL and were all resuspended in 25 μL of 100% methanol. Two μL of the suspensions were injected onto a ACQUITY UPLC system (Waters, Milford, MA) in randomized order with a pooled quality control injection after every six sample injections, and separated using a ACUITY UPLC CSH Phenyl Hexyl column (1.7 μM, 1.0 x 100 mm) (Waters), using a gradient from solvent A (A) (2 mM ammonium hydroxide, 0.1% formic acid) to solvent B (B) (99.9% acetonitrile, 0.1% formic acid). Injections were made in 100% A, held at 100% A for 1 min, ramped to 98% B over 12 min, held at 98% B for 3 min, and then returned to start conditions over 0.05 min and allowed to re-equilibrate for 3.95 min, with a 200 μL/min constant flow rate. The column and samples were held at 65°C and 6°C, respectively. The column eluent was infused into a Xevo G2 Q-TOF-MS (Waters) with an electrospray source in positive mode, scanning 50–2000 m/z at 0.2 s per scan, alternating between MS (6 V collision energy) and MS^E^ mode (15–30 V ramp). Calibration was performed using sodium iodide with 1 ppm mass accuracy. The capillary voltage was held at 2,200 V, source temperature at 150°C, and nitrogen desolvation temperature at 350°C with a flow rate of 800 L/h.

For GC-MS analysis, the extract was resuspended in pyridine containing 25 mg/mL of methoxyamine hydrochloride (5 μL per 5 μg/μL of protein content for granulosa and cumulus cells samples and 25 μL for oocytes), incubated at 60°C for 60 min, vigorously vortexed for 30 s, sonicated for 10 min, and incubated for an additional 60 min at 60°C. Additions of the same volume of N-methyl-N-trimethylsilyltrifluoroacetamide with 1% trimethylchlorosilane (MSTFA +1% TMCS, Thermo Fisher) were made, and samples were vigorously vortexed for 30 s, then incubated at 60°C for 30 min. Metabolites were detected using a TRACE 1,310 GC coupled to a ISQ™ mass spectrometer (ThermoFisher). One μL of the samples were injected at 10:1 split ratio to a 30 m TG-5MS column (Thermo Fisher, 0.25 mm i.d., 0.25 μm film thickness) with a 1.2 mL/min helium gas flow rate. The GC inlet was held at 285°C. The oven program started at 80°C for 30 s, followed by a ramp of 15 ^°^C/min to 330°C, and an 8 min hold. Masses between 50 and 650 m/z were scanned at 5 scans/s under electron impact ionization. Transfer line and ion source were held at 300°C and 260°C, respectively. Pooled quality control samples were injected after every 10 actual samples.

### Data acquisition and analysis

For each sample, raw data files were converted to a computable document format (.cdf), and matrix of molecular features, as defined by retention time and mass (m/z), was generated using XCMS in R software (BMC Bioinformatics) for feature detection and alignment (Smith et al., 2006). Peak areas were also subsequently quantile normalized in R. Outlier injections were detected based on total signal and PC1 of principle component analysis. Features were grouped using RAMClustR (Broeckling et al., 2014), which groups features into spectra based coelution and covariance across the full dataset, whereby spectra are used to determine the identity of observed compounds in the experiment. The peak areas for each feature in a spectrum were condensed via the weighted mean of all features in a spectrum into a single value for each compound. Peak areas are reported by the mass spectrometer in arbitrary units, not in actual concentration units, and are thus described as relative abundance of each metabolite. Compounds were annotated based on spectral matching to in-house, NISTv14, Golm, HMDB and LipidMaps 1-SToP libraries (Broeckling et al., 2016), and Metlin metabolite databases. Heatmaps, volcano plots and PCA plots were created in MetaboAnalyst 5.0. For this, GC-MS and LC-MS datasets were concatenated together, and unit variance scaling was applied to ensure signal intensities from the two platforms were in the same scale for generation of the PCA plots. Normalized abundance of a few metabolites (glucose, pyruvate, lactate, total free fatty acids and total triacylglycerols) in oocytes and cumulus cells at the 42 h maturation time point have been previously reported (Catandi et al., 2021).

### Statistical analyses

All statistical analyses were carried out separately for the data from the cumulus, granulosa and oocyte cell types using R-project software (R Core Team, 2021). Data are presented as $\bar{y}\pm S_{\bar{y}}$ (mean ± standard error of the mean) for mare age and time. For all data sets from the cumulus, granulosa and oocyte cell types, the independent Student’s t-test with the Bonferroni adjustment factor was conducted to evaluate the difference between the means of young and old mares for each time point (0, 24 and 42 h).

Linear relationships were also investigated between the pairwise observations obtained from the cumulus, granulosa and oocyte cell types for all traits in each age group by estimating the Pearson’s correlation coefficients ( $r_{y_{i},y_{j}}$ ) between the pairwise observations ( $y_{i}=y_{cumulus},y_{granulosa},y_{oocyte}$ and $y_{j}\neq y_{i}$ ) from the cumulus, granulosa and oocyte cell types

$$r_{y_{i},y_{j}}=\frac{S_{y_{i},y_{j}}}{\sqrt{S_{y_{i}}^{2}S_{y_{j}}^{2}}}$$

where $S_{y_{i},y_{j}}$ is the covariance between the pairwise observations ( $y_{i}=y_{cumulus},y_{granulosa},y_{oocyte}$ and $y_{j}\neq y_{i}$ ) from cumulus, granulosa and oocyte cell types for the specific trait, $S_{y_{i}}^{2}$ and $S_{y_{j}}^{2}$ are the variances for the pairwise observations from the i and j cell types for the specific trait, respectively. Correlation coefficients (R) between 0.70 and 0.89 were considered indicative of strong correlations, while R > 0.9 was indicative of a very strong correlation (Schober et al., 2018). Differences were considered of significance at *p* < 0.1, with *p*-values for individual metabolites included in tables.

## Results

### Effects of maternal age on the global metabolome

**FIGURE 1:**
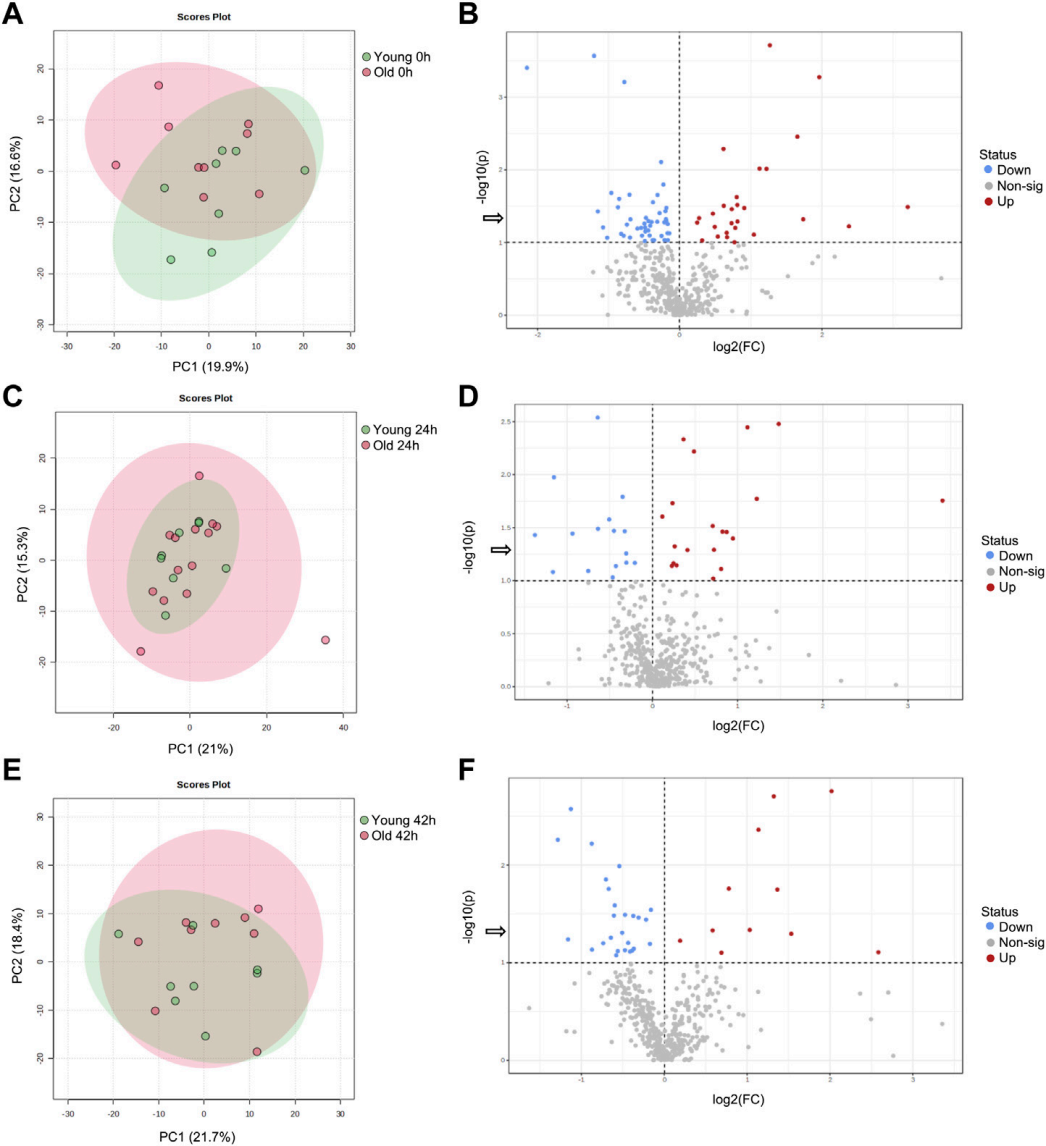
PCA score plots depicting the trend of separation of mare oocyte metabolites between Old (red) and Young (green) at **(A)** 0 h, **(C)** 24 h, and **(E)** 42 h of maturation. Volcano plots depicting metabolites that were reduced (blue) or increased (red) in oocytes from Old in comparison to Young (horizontal dotted line represents *p* < 0.1, arrow represents *p* < 0.05, Student’s *t*-test) at **(B)** 0 h, **(D)** 24 h, and **(F)** 42 h of maturation.

**FIGURE 2:**
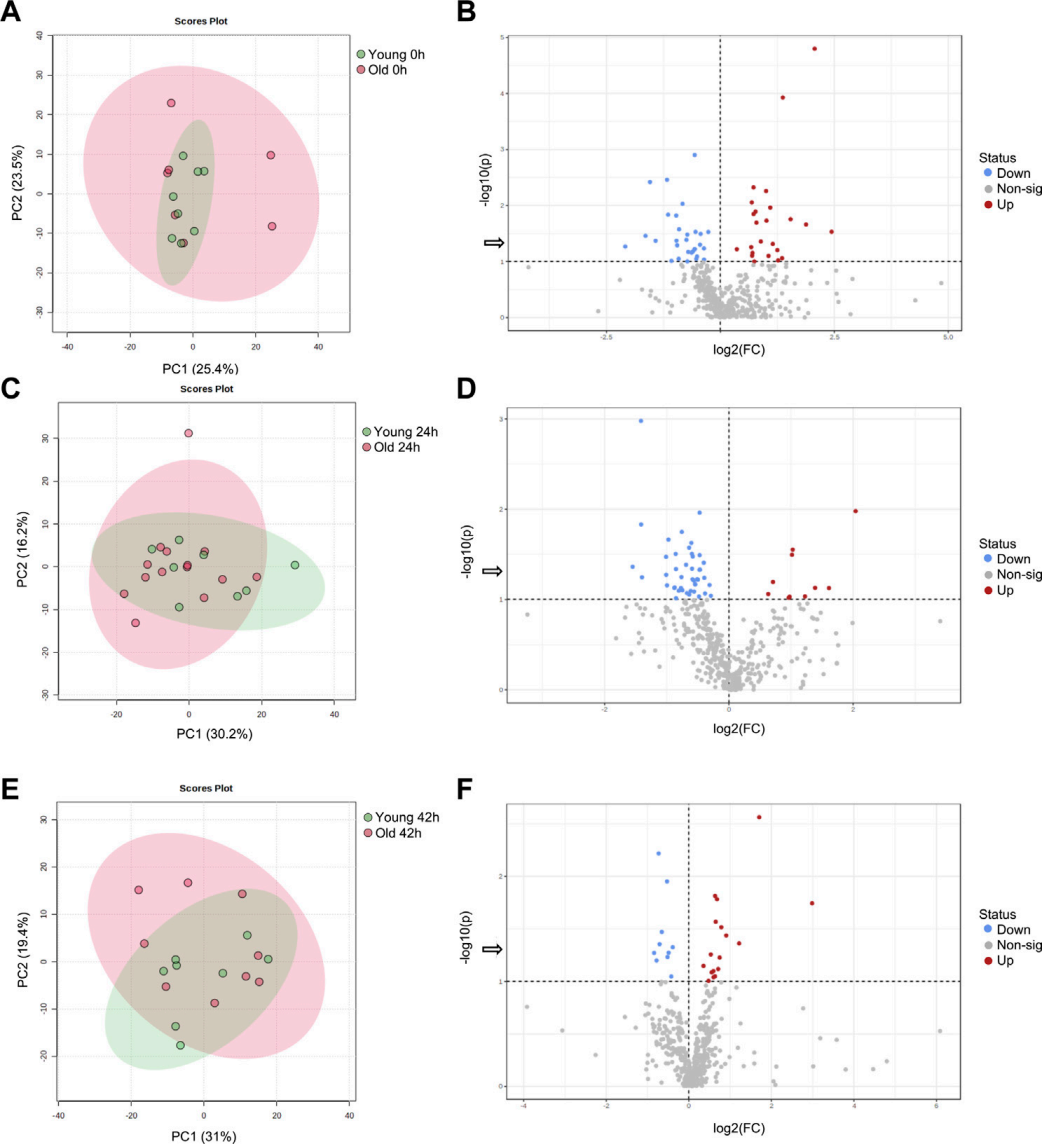
PCA score plots depicting the trend of separation of mare cumulus cell metabolites between Old (red) and Young (green) at **(A)** 0 h, **(C)** 24 h, and **(E)** 42 h of maturation. Volcano plots depicting metabolites that were reduced (blue) or increased (red) in cumulus cells from Old in comparison to Young (horizontal dotted line represents *p* < 0.1, arrow represents *p* < 0.05, Student’s *t*-test) at **(B)** 0 h, **(D)** 24 h, and **(F)** 42 h of maturation.

**FIGURE 3:**
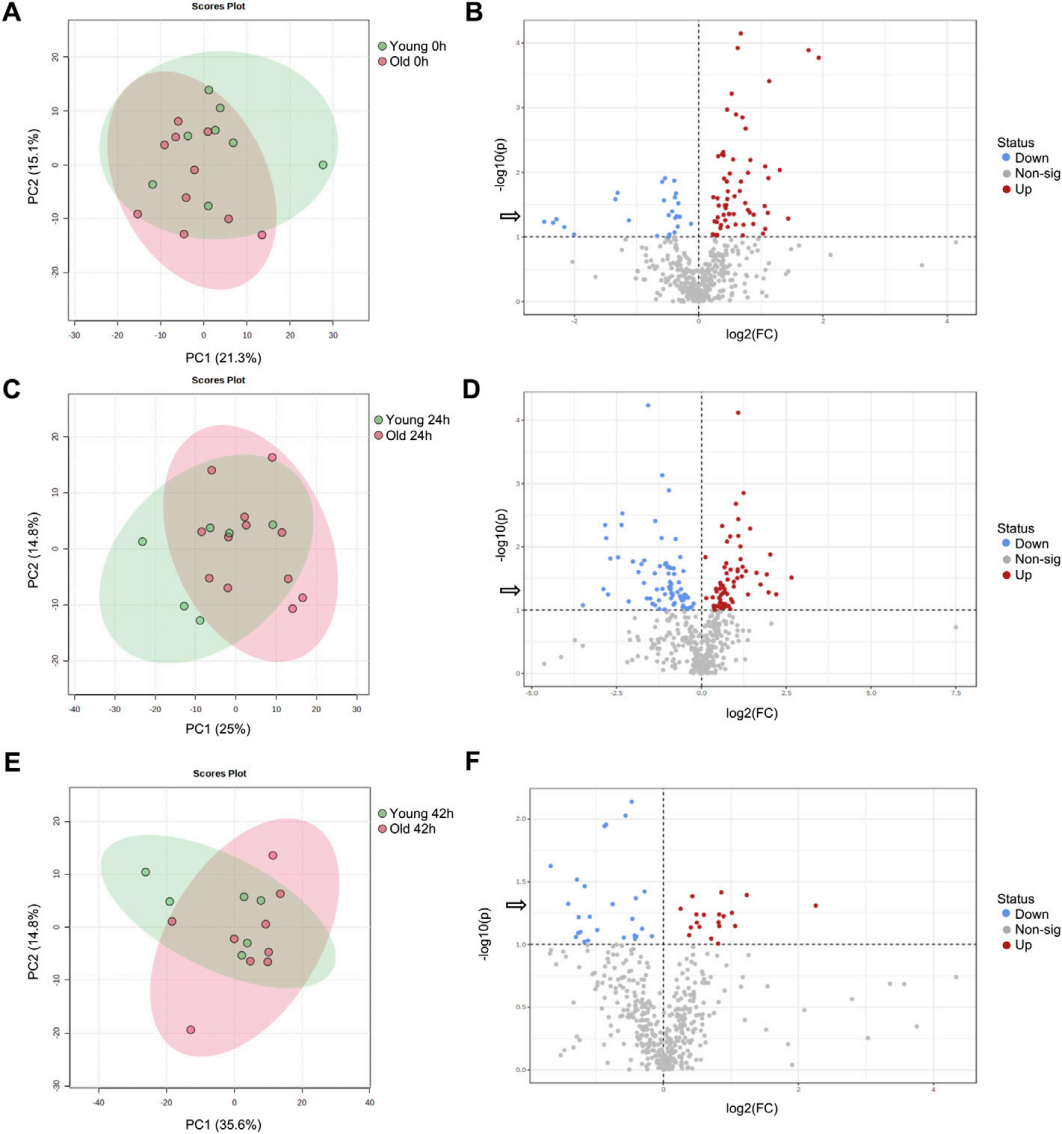
PCA score plots depicting the trend of separation of mare granulosa cell metabolites between Old (red) and Young (green) at **(A)** 0 h, **(C)** 24 h, and **(E)** 42 h of maturation. Volcano plots depicting metabolites that were reduced (blue) or increased (red) in granulosa cells from Old in comparison to Young mares (horizontal dotted line represents *p* < 0.1, arrow represents *p* < 0.05, Student’s *t*-test) at **(B)** 0 h, **(D)** 24 h, and **(F)** 42 h of maturation.

**FIGURE 4:**
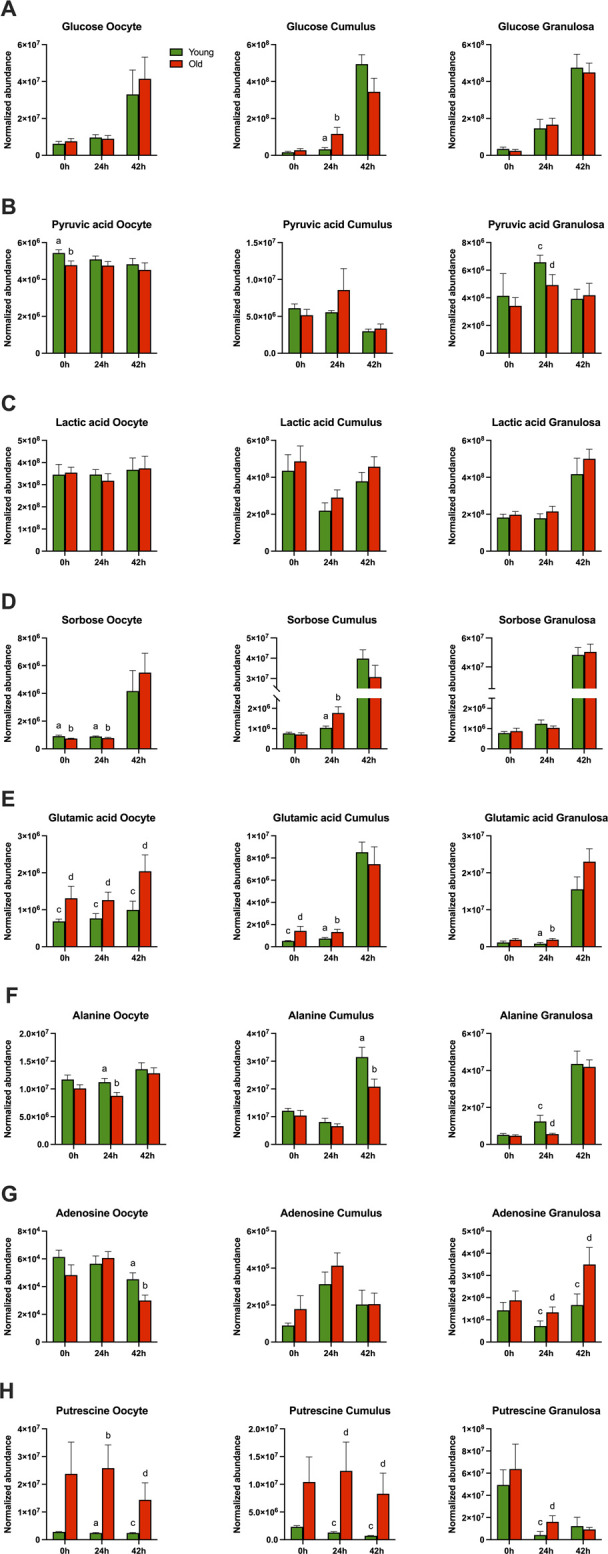
Normalized abundance of main carbohydrates and derivatives and amino acids affected by mare maternal aging in oocytes (first column), cumulus cells (second column) and granulosa cells (third column) at 0h, 24h, and 42 h of maturation: **(A)** glucose, **(B)** pyruvic acid, **(C)** lactic acid, **(D)** sorbose, **(E)** glutamic acid, **(F)** alanine, **(G)** adenosine, and **(H)** putrescine. Bars (green for Young and red for Old) represent mean ± SEM. Superscripts indicate group differences at *p* < 0.05 (ab) and *p* < 0.1 (cd) at each time point (0, 24 or 42 h) obtained using Student’s t tests.

**FIGURE 5:**
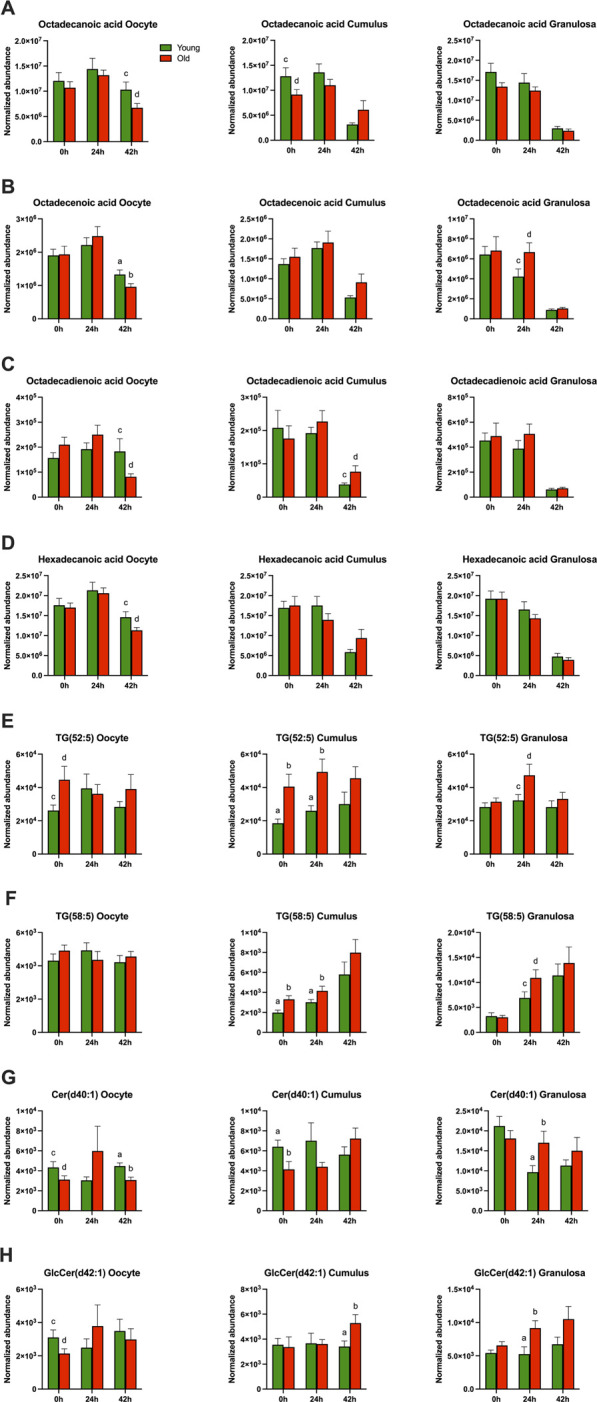
Normalized abundance of main free fatty acids, triacylglycerols, and ceramides affected by mare maternal aging in oocytes (first column), cumulus cells (second column) and granulosa cells (third column) at 0h, 24h, and 42 h of maturation: **(A)** octadecanoic acid, **(B)** octadecenoic acid, **(C)** octadecadienoic acid, **(D)** hexadecenoic acid, **(E)** TG(52:5), **(F)** TG(58:5), **(G)** Cer(d40:1), and **(H)** GlcCer(d42:1). Bars (green for Young and red for Old) represent mean ± SEM. Superscripts indicate group differences at *p* < 0.05 (ab) and *p* < 0.1 (cd) at each time point (0, 24 or 42 h) obtained using Student’s t tests.

From a total of 156 metabolites identified through GC-MS, 30 were annotated. From 297 metabolites identified by LC-MS, 164 were annotated. Metabolite abundance varied among mares, as observed on heatmaps for oocytes (Supplementary Figure S1), cumulus cells (Supplementary Figure S2), and granulosa cells (Supplementary Figure S3) at 0, 24 and 42 h. Although no clear separation was observed between age groups in PCA plots for oocytes (Figures 1A, C, E), cumulus cells (Figures 2A, C, E), and granulosa cells (Figures 3A, C, E), volcano plots demonstrated that some metabolites were significantly affected by mare age for oocytes (Figures 1B, D, F), cumulus cells (Figures 2B, D, F), and granulosa cells (Figures 3B, D, F). All annotated metabolites that were affected by mare age at different maturation time points in oocytes (Supplementary Table S1), cumulus cells (Supplementary Table S2) and granulosa cells (Supplementary Table S3) are included as Supporting Material. Selected glycolytic substrates and amino acids, associated with pathways of interest, that were affected by mare age at 0, 24, or 42 h are illustrated in Figure 4, and selected free fatty acids, triacyclglycerols and ceramides are illustrated in Figure 5.

### Effects of mare age at 0 h (germinal vesicle stage)

In germinal vesicle stage oocytes (0 h) collected without maturation induction, a total of 21 out of 194 (11%) annotated and 46 out of 259 (18%) non-annotated metabolites were affected by mare age (Table 1; Supplementary Table S1). Among polar metabolites, phosphoric acid, glutamic acid (Figure 4E), and β-sitosterol were more abundant in oocytes from old than young mares (*p* ≤ 0.09), while sorbose (Figure 4D), xylose, pyruvic acid (Figure 4B) and glycine were less abundant in oocytes from old than young mares (*p* ≤ 0.05). Among lipid metabolites, two diacylglycerols and one triacylglycerol (Figure 5E) were more abundant (*p* ≤ 0.09), and two diacylglycerols, two phospholipids, and four ceramides (Figures 5G, H) were less abundant (*p* ≤ 0.09) in oocytes from old than young mares.

**TABLE 1:** Relative abundance of selected metabolites in oocytes from young and old mares. Single oocytes were analyzed from Young (*n* = 8 at 0, 24 and 42 h) and Old (0 h, *n* = 10; 24 h, *n* = 14; 42 h, *n* = 9). Results are presented as mean ± SEM, with correction factor of 10^x^ (CF) and *p*-value.

Maturation time (h)	Metabolite class	Metabolite	Young	Old	CF	*p*-value
0	Carbohydrates and derivatives	Sorbose	9.15 ± 0.71	7.40 ± 0.33	10^5^	0.05
		Pyruvic acid	5.43 ± 0.18	4.77 ± 0.23	10^6^	0.04
		Xylose	9.08 ± 0.54	7.60 ± 0.42	10^5^	0.05
	Lipids	TG(52:5)	2.61 ± 0.33	4.46 ± 0.81	10^4^	0.06
		Cer(d40:1)	4.34 ± 0.57	3.11 ± 0.38	10^3^	0.09
		PE(O-36:2)	9.66 ± 1.75	5.29 ± 0.51	10^3^	0.04
	Amino acids	Glutamic acid	0.68 ± 0.06	1.31 ± 0.32	10^6^	0.09
		Glycine (2TMS)	7.31 ± 0.25	6.21 ± 0.27	10^7^	0.01
	Miscellaneous	Phosphoric acid	2.58 ± 0.37	3.93 ± 0.42	10^7^	0.03
		Beta-sitosterol	0.73 ± 0.18	1.27 ± 0.18	10^4^	0.05
24	Carbohydrates	Sorbose	8.88 ± 0.44	7.76 ± 0.44	10^5^	0.09
	Lipids	PC(30:0)	3.72 ± 0.46	6.29 ± 1.35	10^3^	0.09
	Amino acids	Glutamic acid	0.77 ± 0.13	1.26 ± 0.21	10^6^	0.07
		Glycine (3TMS)	2.71 ± 0.07	2.94 ± 0.06	10^7^	0.02
		Alanine	1.12 ± 0.07	0.88 ± 0.06	10^7^	0.02
	Miscellaneous	Putrescine	0.24 ± 0.02	2.58 ± 0.84	10^7^	0.02
		2-hydroxypyridine	4.17 ± 0.32	5.08 ± 0.27	10^8^	0.04
42	Lipids	Octadecanoic acid	1.03 ± 0.15	0.67 ± 0.08	10^7^	0.07
		Octadecenoic acid	1.33 ± 0.14	0.96 ± 0.09	10^6^	0.04
		Octadecadienoic acid	1.83 ± 0.51	0.82 ± 0.12	10^5^	0.09
		Hexadecanoic acid	1.46 ± 0.14	1.13 ± 0.07	10^7^	0.05
	Amino acids	Glutamic acid	1.00 ± 0.24	2.04 ± 0.44	10^6^	0.06
		Glycine (2TMS)	7.37 ± 0.26	6.33 ± 0.37	10^7^	0.04
		Glycine (3TMS)	3.02 ± 0.17	3.55 ± 0.25	10^7^	0.09
	Miscellaneous	Putrescine	0.24 ± 0.02	1.44 ± 0.61	10^7^	0.08
		Beta-sitosterol	0.64 ± 0.18	1.65 ± 0.32	10^4^	0.02
		Adenosine	4.53 ± 0.46	3.00 ± 0.39	10^4^	0.02

In cumulus cells at 0 h, ten out of 194 (5%) annotated and 34/259 (13%) non-annotated metabolites were affected by mare age (Table 2; Supplementary Table S2). Similar to observations for oocytes at 0 h, glutamic acid abundance was higher (Figure 4E; *p* = 0.06) and glycine abundance was lower (*p* = 0.02) in cumulus cells from old than young mares. Additionally, octadecanoic acid (Figure 5A) and cysteine were less abundant in cumulus cells from old than young mares (*p* ≤ 0.09). For lipids, three triacylglycerols were more abundant (Figures 5E, F; *p* ≤ 0.04), while two phospholipids and one ceramide (Figure 5G) were less abundant (*p* ≤ 0.04) in cumulus cells from old than young mares. One triacylglycerol [TG(52:5)], one phospholipid [PE(O-36:2)] and one ceramide [Cer(d40:1)] were similarly affected by mare age in oocytes and cumulus cells.

**TABLE 2:** Relative abundance of main annotated metabolites in cumulus cells affected by mare age. Cumulus cells were analyzed from Young (0, 24, 42 h *n* = 8) and Old (0 h *n* = 8, 24 h *n* = 14, 42 h *n* = 10). Results are presented as mean ± SEM, correction factor of 10^x^ (CF), and *p*-value.

Maturation time (h)	Metabolite class	Metabolite	Young	Old	CF	*p*-value
0	Lipids	Octadecanoic acid	1.28 ± 0.17	0.91 ± 0.10	10^7^	0.09
		TG(52:5)	1.85 ± 0.26	4.05 ± 0.74	10^4^	0.02
		TG(58:5)	1.99 ± 0.24	3.31 ± 0.36	10^3^	0.01
		TG(62:14)	1.15 ± 0.15	2.42 ± 0.51	10^3^	0.04
		Cer(d40:1)	6.41 ± 0.66	4.15 ± 0.77	10^3^	0.04
		PE(O-36:2)	7.83 ± 1.12	4.77 ± 0.71	10^3^	0.04
	Amino acids	Glutamic acid	0.52 ± 0.04	1.43 ± 0.41	10^6^	0.06
		Glycine (2TMS)	6.76 ± 0.54	4.98 ± 0.45	10^7^	0.02
		Cysteine	1.75 ± 0.27	1.21 ± 0.10	10^5^	0.09
24	Carbohydrates	Glucose	0.33 ± 0.09	1.16 ± 0.35	10^8^	0.04
		Sorbose	1.04 ± 0.08	1.77 ± 0.31	10^6^	0.04
	Lipids	TG(52:5)	2.60 ± 0.31	4.93 ± 0.78	10^4^	0.01
		TG(56:7)	1.82 ± 0.32	3.51 ± 0.63	10^4^	0.03
		TG(58:6)	5.27 ± 1.07	9.37 ± 1.35	10^3^	0.03
		TG(58:8)	2.48 ± 0.34	3.73 ± 0.45	10^4^	0.04
		TG(54:7)	1.14 ± 0.24	2.29 ± 0.44	10^4^	0.03
		TG(52:5)	2.01 ± 0.40	4.49 ± 0.81	10^4^	0.01
		TG(56:8)	1.19 ± 0.35	2.56 ± 0.46	10^4^	0.03
		TG(58:5)	3.02 ± 0.27	4.16 ± 0.46	10^3^	0.05
		TG(56:5)	2.05 ± 0.30	3.11 ± 0.36	10^4^	0.04
		TG(54:8)	2.41 ± 0.47	6.04 ± 1.24	10^3^	0.01
		PC(30:0)	2.01 ± 0.33	3.42 ± 0.50	10^4^	0.03
	Amino acids	Glutamic acid	0.74 ± 0.09	1.33 ± 0.23	10^6^	0.03
		Pyroglutamic acid	0.55 ± 0.07	1.35 ± 0.42	10^7^	0.08
		Glycine (2TMS)	6.69 ± 0.32	4.74 ± 0.50	10^7^	0.004
	Miscellaneous	Phosphoric acid	0.51 ± 0.10	1.06 ± 0.19	10^8^	0.02
		Myo-inositol	2.56 ± 0.46	7.02 ± 2.08	10^6^	0.05
42	Lipids	Octadecadienoic acid	3.81 ± 0.46	7.64 ± 1.79	10^4^	0.06
		TG(56:5)	4.53 ± 0.73	5.59 ± 0.62	10^4^	0.05
	Amino acids	Alanine	3.15 ± 0.35	2.08 ± 0.28	10^7^	0.03
	Miscellaneous	Putrescine	0.71 ± 0.05	8.30 ± 3.71	10^6^	0.07

In granulosa cells at 0h, 23/194 (12%) annotated and 36/259 (14%) non-annotated metabolites were affected by mare age (Table 3; Supplementary Table S3). Most were lipids; the exception was glycine, which was more abundant (*p* = 0.05) in granulosa cells from old when compared to young mares. Among differing lipids between the age groups, most were phospholipids; nine were more abundant (*p* ≤ 0.09), and four were less abundant (*p* ≤ 0.05) in old than young. One sphingomyelin and three diacylglycerol species were higher in abundance in granulosa cells of old than young mares. Similar to oocytes, DG(O-36:4) was less abundant in granulosa cells from old than young mares (*p* = 0.04).

**TABLE 3:** Relative abundance of main annotated metabolites in granulosa cells affected by mare age. Granulosa cells were analyzed from Young (0 h *n* = 8, 24 h, 42 h *n* = 6) and Old (0 h *n* = 11, 24 h *n* = 15, 42 h *n* = 9). Results are presented as mean ± SEM, correction factor of 10^x^ (CF), and *p*-value.

Maturation time (h)	Metabolite class	Metabolite	Young	Old	CF	*p*-value
0	Lipids	DG(36:4)	2.68 ± 0.46	4.05 ± 0.33	10^4^	0.03
		DG(O-36:4)	2.34 ± 0.20	1.59 ± 0.27	10^3^	0.04
		DG(38:4)	0.84 ± 0.13	1.12 ± 0.87	10^5^	0.09
	Amino acids	Glycine (3TMS)	2.35 ± 0.27	3.02 ± 0.15	10^7^	0.05
24	Carbohydrate derivatives	Pyruvic acid	6.57 ± 0.50	4.92 ± 0.76	10^6^	0.09
	Lipids	Octadecenoic acid	4.22 ± 0.77	6.67 ± 0.92	10^6^	0.06
		Linoleic acid	0.58 ± 0.13	1.02 ± 0.20	10^3^	0.09
		TG(52:5)	3.22 ± 0.35	4.73 ± 0.66	10^4^	0.06
		TG(56:7)	4.09 ± 0.11	5.57 ± 0.49	10^4^	0.07
		TG(54:7)	1.23 ± 0.14	1.93 ± 0.22	10^4^	0.02
		TG(58:5)	0.69 ± 0.12	1.09 ± 0.16	10^4^	0.07
		TG(48:1)	0.85 ± 0.04	1.27 ± 0.16	10^4^	0.03
		TG(56:5)	4.00 ± 0.40	5.78 ± 0.54	10^4^	0.02
		TG(49:1)	2.37 ± 0.22	3.66 ± 0.51	10^3^	0.03
		DG(36:4)	0.58 ± 0.06	1.52 ± 0.33	10^4^	0.01
		DG(38:4)	1.91 ± 0.33	4.50 ± 1.04	10^4^	0.03
		GalCer(d42:2)	5.94 ± 1.31	9.44 ± 1.06	10^3^	0.06
		Cholestenone	2.08 ± 0.17	1.19 ± 0.11	10^4^	0.002
		5a-Cholestanol	2.74 ± 0.58	1.42 ± 0.21	10^3^	0.08
		PC(36:4)	1.85 ± 0.26	2.52 ± 0.27	10^5^	0.09
		PE(42:2)	1.53 ± 0.16	1.92 ± 0.13	10^4^	0.09
		PC(O-36:3)	1.55 ± 0.15	1.12 ± 0.13	10^4^	0.05
		LPC(16:1)	4.42 ± 0.92	2.27 ± 0.40	10^3^	0.07
	Amino acids	Glutamic acid	0.81 ± 0.33	1.90 ± 0.33	10^6^	0.04
		Cysteine	0.28 ± 0.11	1.08 ± 0.25	10^6^	0.01
		Alanine	1.24 ± 0.33	0.55 ± 0.44	10^7^	0.09
		Glycine (3TMS)	2.85 ± 0.38	4.53 ± 0.44	10^7^	0.01
	Miscellaneous	Putrescine	0.42 ± 0.32	1.61 ± 0.55	10^7^	0.07
		Phosphoric acid	1.62 ± 0.45	3.44 ± 0.47	10^8^	0.01
		Adenosine	0.72 ± 0.23	1.34 ± 0.24	10^6^	0.08
		Myo-inositol	4.03 ± 1.42	7.75 ± 1.15	10^6^	0.06
42	Lipids	CE(18:2)	3.91 ± 0.95	1.72 ± 0.36	10^4^	0.06
	Amino acids	Glycine (3TMS)	1.15 ± 0.14	1.59 ± 0.20	10^8^	0.09
	Miscellaneous	Beta-sitosterol	2.66 ± 0.80	4.73 ± 0.78	10^3^	0.09
		Adenosine	1.67 ± 0.50	3.50 ± 0.76	10^6^	0.09

### Effects of mare age at 24 h (metaphase I)

Ten out of 194 (5%) annotated and 28/259 (11%) non-annotated metabolites were affected by mare age in oocytes collected approximately 24 h after ovulation induction and at the anticipated metaphase I stage of development (Table 1; Supplementary Table S1). The only differences observed at 0 h that were maintained at 24 h were higher abundance of glutamic acid (Figure 4E; *p* = 0.02) and lower abundance of sorbose (Figure 4D; *p* = 0.07) in oocytes from old when compared to young mares. Glycine, 2-hydroxy-pyridine and putrescine (Figure 4H) were more abundant (*p* ≤ 0.04) and alanine was less abundant (Figure 4F; *p* = 0.02) in old than young oocytes. Among lipid metabolites, one phospholipid and one ceramide were more abundant (*p* ≤ 0.09) and one diacylglycerol was less abundant (*p* = 0.08) in oocytes from old than young mares.

In cumulus cells, 22/194 (11%) annotated and 37/259 (14%) non-annotated metabolites were affected by mare age at 24 h (Table 2; Supplementary Table S2). Differences that were similar in cumulus cells and oocytes at 24 h were a greater abundance of glutamic acid (Figure 4E) and PC(30:0) in cumulus cells from old when compared to young mares (*p* ≤ 0.03). Interestingly, sorbose followed an opposite trend than observed in oocytes, being more abundant in cumulus cells from old than young mares (Figure 4D; *p* = 0.04). Moreover, myo-inositol, glucose (Figure 4A), phosphoric acid and pyroglutamic acid were all more abundant (*p* ≤ 0.08) and glycine was less abundant in cumulus cells from old than young (*p* = 0.004). For lipid metabolites, most differences were observed in triacylglycerols, with 10 TG species being more abundant (Figures 5E, F; *p* ≤ 0.05) in old than young cumulus cells. One cholesteryl ester and two ceramides were less abundant (*p* < 0.05) in cumulus cells from old than young mares. Differences observed at 0 h that were maintained at 24 h were higher abundance of glutamic acid, TG(52:5) and TG(58:5), and lower abundance of glycine in cumulus cells from old when compared to young mares.

In granulosa cells at 24 h, 56/194 (29%) annotated and 59/259 (23%) non-annotated metabolites were affected by mare age (Table 3; Supplementary Table S3). Some differences were similar as observed in oocytes at 24 h, with a higher abundance of glycine, putrescine (Figure 4H) and GalCer(d42:2) (*p* ≤ 0.07) and lower abundance of alanine (Figure 4F; *p* < 0.1) for old than young mares. Some differences were similar to observed in cumulus cells at 24 h, including a higher abundance of myo-inositol, phosphoric acid, and five triacylglycerols [TG(52:5) (Figure 5E), (56:7), (54:7), (58:5) (Figure 5F) and (56:5)] in granulosa cells from old than young mares (*p* ≤ 0.06). Furthermore, higher abundance of glutamic acid in old than young was observed in oocytes, cumulus and granulosa cells (Figure 4; *p* = 0.04). Among polar metabolites, adenosine, octadecenoic acid (Figure 5B), linoleic acid and cysteine were more abundant (*p* ≤ 0.08) and alanine (Figure 4F) and pyruvic acid (Figure 4B) were less abundant (*p* < 0.1) in granulosa cells from old than young mares. Amidst the many differing lipid species, the main findings were lower abundance of two cholesterol derivatives and five phospholipids (*p* ≤ 0.08) and higher abundance of eight phospholipids, five ceramides, one sphingomyelin, six diacylglycerols and seven triacylglycerols (*p* ≤ 0.09) in old than young mares’ granulosa cells. Differences observed at 0 h that were maintained at 24 h were higher abundance of glycine, PC(36:4), PE(42:2), DG(38:4) and DG(36:4) in granulosa cells from old when compared to young mares.

### Effects of mare age at 42 h (metaphase II)

Fourteen out of 194 (7%) annotated and 24/259 (9%) non-annotated metabolites were affected by mare age in oocytes at the metaphase II stage of development, after collection from the follicle at approximately 24 h after ovulation induction plus 18 h of culture (Table 1; Supplementary Table S1). Differences observed at 24 h that were maintained at 42 h include higher abundance of glutamic acid (Figure 4E), putrescine (Figure 4H), and glycine (*p* ≤ 0.09) in oocytes from old when compared to young mares. Moreover, some differences that were observed at 0 h were also noted at 42 h, with lower glycine and higher β-sitosterol abundance for old than young mares (*p* ≤ 0.04). Age effects that were observed for oocytes, including lower abundances of adenosine (Figure 4G), octadecanoic acid (Figure 5A), octadecenoic acid (Figure 5B), octadecadienoic acid (Figure 5C), hexadecanoic acid (Figure 5D), two phospholipids, and one ceramide in old when compared to young mares (*p* ≤ 0.09).

In cumulus cells, eight out of 194 (4%) annotated and 17/259 (7%) non-annotated metabolites were affected by mare age (Table 2; Supplementary Table S2). Similar to observations for oocytes at 42 h, abundance of putrescine (Figure 4H) and octadecadienoic acid (Figure 5C) were higher (*p* ≤ 0.07) in cumulus cells from old when compared to young mares. Additionally, alanine (Figure 4F) and one phospholipid were less abundant (*p* ≤ 0.04), while one phospholipid, one ceramide (Figure 5H), one diacylglycerol and one triacylglycerol were more abundant in cumulus cells from old than young mares (*p* ≤ 0.05).

In granulosa cells, 11/194 (6%) annotated and 12/259 (5%) non-annotated metabolites were affected by mare age (Table 3; Supplementary Table S3). Some differences were similar as observed in oocytes at 42 h, including a higher abundance of glycine, adenosine (Figure 4G) and β-sitosterol (*p* ≤ 0.09) in granulosa cells from old than young. Three phospholipids and two cholesteryl esters were more abundant; two ceramides, one sphingomyelin and one diacylglycerol were less abundant in granulosa cells from old than young mares. Differences observed at 24 h that were maintained at 42 h were higher abundance of glycine, Cer(42:2) and SM(24:0) in granulosa cells from old than young mares.

### Metabolite correlations between different follicular cell types

**FIGURE 6:**
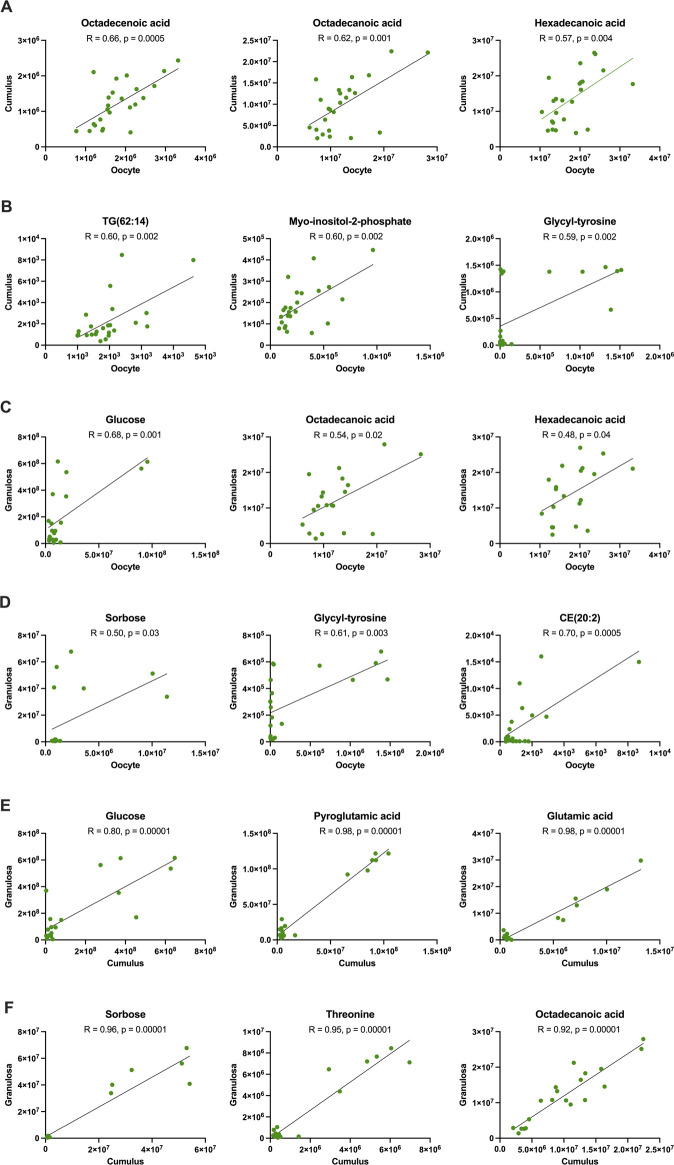
Correlation of metabolites between oocytes and cumulus cells (rows **(A, B)**), oocytes and granulosa cells (rows **(C, D)**), and cumulus and granulosa cells (rows **(E, F)**) collected from young mares, including all maturation times. Correlation coefficients (R) obtained using Pearson’s correlation test.

Among the different follicle cell types from young mares, the strongest correlations were observed between cumulus and granulosa cells (Table 4; Supplementary Table S4). Although significant correlations were observed for metabolites between oocytes and cumulus cells, correlation coefficients were moderate for free fatty acids [octadecenoic (R = 0.66; Figure 6A), octadecanoic (R = 0.62; Figure 6A), and hexadecenoic acids (R = 0.57; Figure 6A)], a triacylglycerol [TG(62:14); R = 0.60; Figure 6B], myo-inositol-2-phosphate (R = 0.60; Figure 6B), and glycyl-tyrosine (R = 0.59; Figure 6B) (*p* ≤ 0.002). Between oocyte and granulosa cell metabolites, one strong correlation was observed for a cholesteryl ester [CE(20:2)] (R = 0.70; *p* = 0.0005; Figure 6D), and glucose was the second strongest correlation observed between these cell types from young mares (R = 0.68; *p* = 0.001; Figure 6C). Several very strong and strong correlations were noted between cumulus and granulosa cell metabolites from young mares. Very strongly correlated metabolites (R ≥ 0.9) were mostly amino acids (pyroglutamic acid, glutamic acid, threonine; Figures 6E, F), monosaccharides (sorbose; Figure 6F), and free fatty acids (octadecanoic acid; Figure 6F) (*p* = 0.00001); strong correlations (R ≥ 0.71) included amino acids (alanine, glycine, serine, cysteine, adenosine), glucose (R = 0.80; Figure 6E), free fatty acid (hexadecanoic acid), myo-inositol, cholesteryl esters [CE(20:2) and CE(22:5)], and triglycerides [TG(56:5) and TG(58:6)] (*p* ≤ 0.0006).

**TABLE 4:** Selected metabolites with correlation coefficients (R) and *p*-values among oocytes, cumulus cells, and granulosa cells in young mares.

	Oocyte and cumulus cells (n = 24)		Oocyte and granulosa cells (*n* = 21)		Cumulus and granulosa cells (*n* = 20)	
Metabolite	R	*p*-value	R	*p*-value	R	*p*-value
Octadecenoic acid	0.7	0.0005	0.3	0.2	0.5	0.04
Octadecanoic acid	0.6	0.001	0.5	0.02	0.9	0.00001
Octadecadienoic acid	0.02	0.9	−0.2	0.5	0.5	0.04
Hexadecanoic acid	0.6	0.004	0.5	0.04	0.8	0.0001
TG(62:14)	0.6	0.002	0.3	0.2	0.6	0.007
TG(58:9)	0.5	0.02	0.3	0.2	0.5	0.01
TG(56:7)	0.2	0.4	0.4	0.09	0.7	0.0007
CE(20:2)	0.5	0.009	0.7	0.0005	0.9	0.00001
Glucose	0.3	0.2	0.7	0.001	0.8	0.00001
Sorbose	0.3	0.1	0.5	0.03	0.9	0.00001
Phosphoric acid	0.4	0.04	0.3	0.2	0.1	0.7
Glutamic acid	0.1	0.7	0.1	0.7	0.9	0.00001
Pyroglutamic acid	0.2	0.3	0.4	0.1	0.9	0.00001
Glycyl-tyrosine	0.6	0.002	0.6	0.003	0.6	0.008
Threonine	0.1	0.5	0.2	0.4	0.9	0.00001
Alanine	0.2	0.3	0.2	0.1	0.9	0.00001
Serine	0.1	0.7	0.3	0.3	0.8	0.00001
Glycine	0.2	0.4	0.2	0.3	0.9	0.00001
Cysteine	−0.3	0.1	−0.2	0.3	0.7	0.0006
Adenosine	0.2	0.4	0.1	0.6	0.8	0.00001
Putrescine	0.2	0.4	0.3	0.2	0.4	0.05
Myo-inositol	−0.004	0.99	−0.1	0.7	0.8	0.0001
Myo-inositol-2P	0.6	0.002	0.3	0.2	0.04	0.8

**FIGURE 7:**
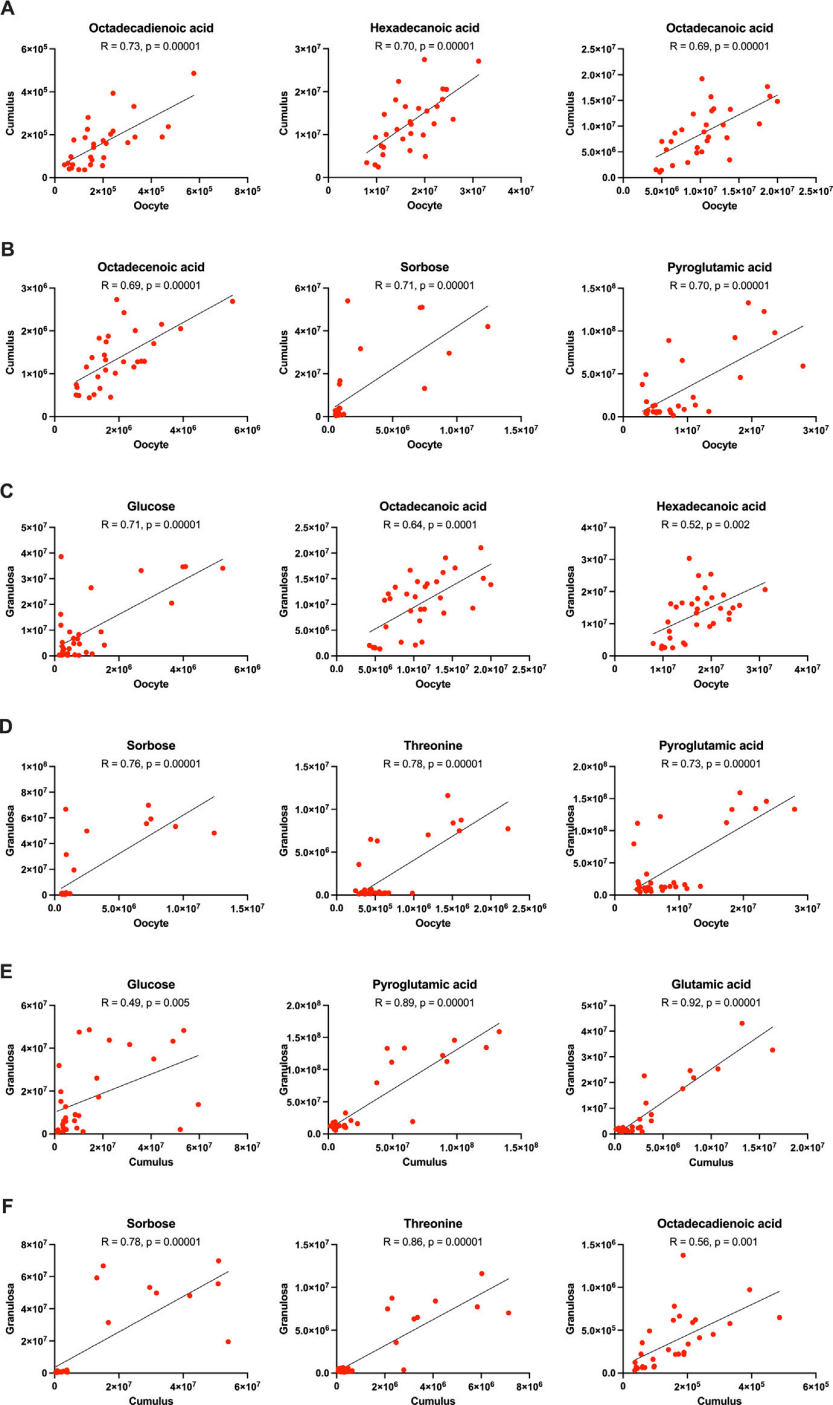
Correlation of metabolites between oocytes and cumulus cells (rows **(A, B)**), oocytes and granulosa cells (rows **(C, D)**), and cumulus and granulosa cells (rows **(E, F)**) collected from old mares, including all maturation times. Correlation coefficients (R) obtained using Pearson’s correlation test.

For old mares, a few strong correlations (R ≥ 0.70) were observed between oocyte and cumulus cell metabolites (Table 5; Supplementary Table S5), including free fatty acids (octadecadienoic and hexadecenoic acids; Figure 7A), sorbose (Figure 7B), pyroglutamic acid (Figure 7B) and one diacylglycerol [DG(40:8)] (*p* = 0.00001). Metabolites that correlated strongly (R ≥ 0.71) between oocytes and granulosa cells from old mares included monosaccharides (glucose and sorbose; Figures 7C, D), threonine (Figure 7D) and pyroglutamic acid (*p* = 0.00001; Figure 7D). A very strong correlation (R = 0.92) was observed for glutamic acid between cumulus and granulosa cells from old mares (*p* = 0.00001; Figure 7E); but several other metabolites were strongly correlated (R ≥ 0.70) between these cell types, including amino acids [threonine (Figure 7F), glycine, alanine, glycyl-tyrosine, adenosine], pyroglutamic acid (Figure 7E), sorbose (Figure 7F), one cholesteryl ester [CE(20:2)], one diacylglycerol [DG(40:8)], and three phospholipids [PC(28:0), PC(39:1) and PE(42:0)].

**TABLE 5:** Selected metabolites with correlation coefficients (R) and *p*-values among oocytes, cumulus cells, and granulosa cells in old mares.

	Oocyte and cumulus cells (*n* = 30)		Oocyte and granulosa cells (*n* = 33)		Cumulus and granulosa cells (*n* = 30)	
Metabolite	R	*p*-value	R	*p*-value	R	*p*-value
Octadecenoic acid	0.7	0.00001	0.4	0.02	0.4	0.03
Octadecanoic acid	0.7	0.00001	0.6	0.0001	0.5	0.01
Octadecadienoic acid	0.7	0.00001	0.6	0.001	0.2	0.2
Hexadecanoic acid	0.7	0.00001	0.5	0.007	0.5	0.002
TG(62:14)	0.2	0.4	0.1	0.7	0.5	0.002
TG(58:9)	0.3	0.08	0.2	0.2	0.6	0.0006
TG(56:7)	0.3	0.1	0.02	0.9	0.4	0.03
CE(20:2)	0.3	0.1	0.2	0.2	0.8	0.00001
Glucose	0.6	0.001	0.7	0.00001	0.5	0.005
Sorbose	0.7	0.00001	0.8	0.00001	0.8	0.00001
Glutamic acid	0.5	0.004	0.4	0.009	0.9	0.00001
Pyroglutamic acid	0.7	0.00001	0.7	0.00001	0.9	0.00001
Glycyl-tyrosine	0.5	0.007	0.4	0.02	0.8	0.00001
Threonine	0.7	0.00001	0.8	0.00001	0.9	0.00001
Alanine	0.6	0.001	0.6	0.0002	0.8	0.00001
Serine	0.1	0.7	0.4	0.04	0.5	0.007
Glycine	0.6	0.0002	0.5	0.0008	0.8	0.00001
Cysteine	−0.2	0.2	−0.4	0.04	0.5	0.009
Adenosine	0.6	0.0009	0.4	0.03	0.7	0.00001
Putrescine	0.4	0.03	−0.2	0.3	−0.1	0.5
Myo-inositol	0.5	0.003	0.5	0.002	0.5	0.002
Myo-inositol-2P	0.3	0.07	0.4	0.01	0.35	0.06

## Discussion

Oocyte quality has been shown to be an essential component of fertility in many mammalian species. The oocyte must obtain factors essential for fertilization and early embryo development prior to ovulation (Zhu and Wang, 2022), and somatic cells within the ovarian follicle are integral to this process. In general, granulosa cells shuffle systemic compounds into the follicular fluid, and cumulus cells help to uptake, convert, and transport compounds to the oocyte (Turathum et al., 2021). The decline in oocyte quality with advanced maternal aging is well-documented in horses, women, and many other species (Carnevale and Ginther, 1995; Krisher, 2019a). However, mechanisms behind the loss of oocyte quality with maternal aging are not fully understood and are likely multifactorial, complex, and associated with alterations in the follicular somatic cells.

The metabolome of the granulosa cells, cumulus cells, and the oocyte provides a window into the physiological status of the follicle, which could deliver insight into causes of age-associated reproductive dysfunction and a basis for potential biomarkers and therapeutic interventions. In the present study, the metabolome of follicular cells and oocytes were compared between young and old mares at three specific stages of maturation. To provide consistency among the samples, cells were collected only from large, dominant follicles during the follicular phase of the cycle, representing the follicles that would be destined for ovulation. Samples at 0 and 24 h were consistent with the germinal vesicle and metaphase I stages of oocyte maturation, respectively, while 42 h samples were collected 24 h after maturation induction with an additional 18 h of culture for the completion of maturation of the oocyte to metaphase II. The 42 h samples are consistent with those used in our laboratory to produce embryos and foals after oocyte transfer or intracytoplasmic sperm injection (ICSI) confirming the suitability of the culture conditions (Frank et al., 2019; Catandi et al., 2021). This study highlights differences in metabolites involved with glucose, amino acid, and lipid metabolism between age groups at the three stages of follicle/oocyte maturation and aims to help answer the hypothesis that metabolic dysfunction of cells within the follicle contributes to a loss in reproductive performance with ageing.

Oocyte developmental potential relies on energy-demanding events for maturation, fertilization, and early embryo development. Mitochondrial respiration is the most efficient method of energy production; however, a balance between aerobic and anaerobic metabolism is critical for development of the oocyte and early embryo (May-Panloup et al., 2007; Adhikari et al., 2022). During oocyte maturation, mitochondria replicate and increase in number in preparation for cleavage divisions (Babayev and Seli, 2015). This step is crucial for the success of embryo development, as mitochondrial replication is arrested beginning at early cleavage stages and until blastocyst formation the equine and other species (Babayev and Seli, 2015; Hendriks et al., 2019). We previously demonstrated that mare advanced aging negatively affects the aerobic and anaerobic metabolic rate and capacity of oocytes, limiting oocyte energy production and developmental potential (Catandi et al., 2019; 2020; 2021). Results obtained in the present study, regarding metabolomic composition of oocytes and follicular cells from old and young mares, further suggest that several metabolic pathways are affected by maternal aging.

During oocyte development and maturation, cumulus cells metabolize glucose and transfer the resulting pyruvate or lactate to oocytes via transzonal projections and gap junctions (Herrick et al., 2017). Glucose is generally not considered important for oocyte energy production because of limited phosphofructokinase expression in oocytes when compared to cumulus cells (Richani et al., 2021); this was initially demonstrated in the bovine (Cetica et al., 2002), although species differences may exist. However, direct transport of glucose to the oocyte is important for other pathways, especially the pentose phosphate pathway, which mediates nuclear maturation and redox balance (Cetica et al., 2002; Krisher, 2019b). Equine cumulus oocyte complexes consume more glucose at 10–20 h of *in vitro* maturation than cumulus cells alone (Lewis et al., 2020), suggesting some glucose is directly transferred to the oocyte and more glucose is converted to other substrates for the oocyte. During maturation, most glucose consumed by equine cumulus oocyte complexes is directed for anaerobic glycolysis and production of lactate (Lewis et al., 2020). In the present study, glucose abundance was greater in the cumulus cells from old mares at 24 h; however, this did not result in increased glucose, pyruvic acid, or lactic acid availability in the oocyte. This observation may indicate impaired transport or production of these key substrates, possibly due to the increased breakdown of transzonal projections with age or metabolic dysfunction. This is supported by lower levels of pyruvic acid in GV stage oocytes in old mares, a primary source of energy for the developing oocyte and early embryo. We did not observe a difference among the age groups in lactic acid for any cell type; although in previous studies, metaphase II oocytes from old mares demonstrated an impaired anaerobic metabolic rate and capacity (Catandi et al., 2021). Taken together, these data suggest that cumulus cells in older mares may increase glucose uptake to combat impaired transport or production of metabolic substrates to the maturing oocyte, or they may be less capable of using the substrates to maintain essential cell functions.

The metabolomic analysis used in this study allowed us to highlight a broad range of metabolites associated with different pathways; many of which have not been considered to play in age-associated ovarian dysfunction and decreased oocyte competence. Although minimally studied in mammalian follicles, sorbose is a ketose belonging to the monosaccharide group of sugars (Wen et al., 2015). In other cell types, such as canine erythrocytes and murine cancer cells, sorbose inhibits hexokinase, thus limiting glycolysis (Kistler and Keller, 1978; Xu et al., 2023). In murine neoplastic cells, sorbose impairs mitochondrial function, stimulates ROS production, and attenuates cellular antioxidant defenses, ultimately contributing to apoptosis (Xu et al., 2023). In the present study. increased sorbose abundance was observed in cumulus cells from old *versus* young mares at metaphase I (24 h). Although the specific role of sorbose in follicular cell metabolism has not been described, increased ROS production and lowered expression of antioxidant enzymes has been demonstrated in oocytes of aged mice (Pasquariello et al., 2019). It is possible that increased sorbose may contribute to these deleterious effects; however, details regarding the source and effect of sorbose in the ovarian follicular environment require further investigations, including the potential significance of age-associated alterations.

Myo-inositol is a derivative of glucose which can mediate post-receptor effects of insulin (Merviel et al., 2021). This carbocyclic sugar has wide-spread roles in cellular signaling and proliferation, as well as antioxidant functions, and its presence has been positively associated with oocyte quality *in vivo* and *in vitro* (Papaleo et al., 2009; Mohammadi et al., 2020). Supplementation of myo-inositol has been used to normalize ovarian function and improve oocyte quality in women with polycystic ovarian syndrome (Merviel et al., 2021) and to improve pregnancy rates in case studies of women, 36–43 years of age, although the women received additional antioxidant and nutrient supplements (Jorge et al., 2023). At the metaphase I stage of oocyte development (24 h), myo-inositol was higher in the cumulus cells of old than young mares. This may have been augmented by increased cumulus cell glucose uptake in old mares during this time, as supported by this study. The specific function of myo-inositol in cumulus cells has not been described, although the promotion of insulin signaling and cell proliferation in cumulus cells is critical to development and may reflect a compensatory mechanism to improve metabolic function in the follicular cells of old mares.

Lipids provide another source of energy for the oocyte and early embryo. The lipid content of oocytes varies among species (Dunning et al., 2014). Equine oocytes have a relatively high lipid content and are thought to utilize lipids as the main substrate for oxidative energy production during maturation (Lewis et al., 2020). Triglycerides are one of the major forms of energy storage within somatic follicular cells and oocytes (Dunning et al., 2014). Studies specifically associating oocyte lipid accumulation with maternal aging are scarce, and the benefits *versus* detriments of lipids potentially vary with species. The relative abundance of triglycerides was similar in the oocytes of old and young mares. However, nine and five triglycerides were significantly increased in relative abundance in the cumulus cells and granulosa cells, respectively, of old *versus* young mares at 24 h, associated with the metaphase I stage of maturation. At the completion of maturation (42 h), oocytes from old mares had less abundant free fatty acids than from young mares, while a trend in the opposite direction occurred in cumulus cells. The limited transport of free fatty acids from cumulus cells to oocytes could occur for old mares if a precocious disruption of transzonal cellular communications occurred, as observed in old female mice (El-Hayek et al., 2018). In bovine cumulus oocyte complexes, fatty acids are transported from cumulus cells to the oocyte by fatty acid binding proteins through transzonal projections (del Collado et al., 2017). Although this transport mechanism has not been demonstrated in equine oocytes, the strong correlations observed in free fatty acid abundance between cumulus cells and oocytes suggests similar direct transport of these molecules occur in mares.

Ceramides are derived from sphingolipids; they are important components of cell and mitochondrial membranes and participate in numerous cellular processes such as differentiation, proliferation, apoptosis, and signaling (Gomez-Larrauri et al., 2020). In oocytes, although specific roles of ceramides are uncertain, ceramides are known to be transported to oocytes from cumulus cells through gap junctions, and ceramides are less abundant in oocytes from aged than young mice, with implications in age-dependent acceleration of oocyte apoptosis (Perez et al., 2005). Additionally, reduced mitochondrial abundance of ceramides in oocytes from old than young female mice seem to negatively impact oocyte mitochondrial functionality (Kujjo and Perez, 2012; Kujjo et al., 2013). Ceramide concentrations have also been reported to be lower in follicular fluid from women with low when compared to normal ovarian reserves and can be used as a biomarker for predicting pregnancy outcomes of *in vitro* fertilization (Timur et al., 2022). In our study, oocytes at the germinal vesicle stage tended to have less abundant ceramides when collected from old *versus* young mares, and similar differences were observed in cumulus cells at the germinal vesicle and metaphase I stages. However, in cumulus cells and granulosa cells at metaphase II (42 h), this pattern was less consistent, with the differential abundance of some ceramides being higher for old than young mares. Lower mitochondrial ceramide abundance seems to be linked to age-induced altered mitochondrial function and structure and may be due to impaired lipid transport or limited mitochondrial synthesis of ceramides, but the exact cellular mechanisms remain to be elucidated (Kujjo et al., 2013; Turksen, 2020). In this study we did not quantify mitochondrial-specific ceramides, so we are unable to link reduced oocyte ceramide abundance with previously reported impaired mitochondrial function in oocytes from old relative to young mares (Catandi et al., 2021). In addition, our observation as to differences in ceramide abundance among cell types and mare ages suggests that the contribution of ceramides as a biomarker for age-associated follicular alterations is still inconclusive and requires further investigation.

Amino acids are essential for proper oocyte development and serve many different purposes, including as substrates for synthesis of proteins, nucleotides, glutathione, hyaluronic acid, signaling molecules, and energy (Collado-Fernandez et al., 2012). Amino acid metabolism has been more extensively studied in developing embryos than in oocytes and follicles (Collado-Fernandez et al., 2012; Krisher, 2019b), and specific effects of maternal aging on follicular amino acid profiles and metabolism are largely undetermined, with few published reports (Babayev and Duncan, 2022). In mice during follicular development, the oocyte regulates amino acid uptake by cumulus and granulosa cells via paracrine factors (Eppig et al., 2005). Well known for its role in anabolic metabolism, glutamic acid is among the most prevalent amino acid in mammalian female fluids (Špirková et al., 2022). Glutamine and glutamate are endogenous glutamic acid derivatives, and the terms are sometimes used interchangeably (Kulkarni et al., 2005; Dutta et al., 2013). Glutamic acid and its derivatives can serve as substrates for synthesis of protein, energy and glutathione, and they can act as signaling molecules by interacting with different cellular membrane receptors (Yoo et al., 2020; Špirková et al., 2022). Age-associated differences were frequently noted for the relative abundance of glutamic acid; differences were observed in all cell types at some time points with the relative abundance consistently greater in samples from old than young mares. Because glutamic acid participates in many cellular processes, elevated glutamic acid in oocytes and follicular cells from old when compared to young mares could indicate impaired protein synthesis, energy metabolism, or antioxidant potential; however, this cannot be extrapolated from this study. Glutamine abundance is greater in germinal vesicle and metaphase I oocytes from older women (35–42 years) than women 23–34 years (Smits et al., 2023). The authors postulated the findings in women were associated with impaired mitochondrial tricarboxylic acid cycle efficiency and reduced glutathione synthesis, promoting mitochondrial dysfunction and oxidative stress in oocytes from advanced maternal age women (Smits et al., 2023). This would be consistent with the altered oocyte metabolic function and positive response to dietary antioxidants that we have observed for old mares in a previous study (Catandi et al., 2021; Catandi et al., 2022). Potentially, the prevalence of glutamic acid can serve as a biomarker for age-associated follicular alterations. An intermediate in glutathione metabolism, pyroglutamic acid, is inversely proportionate to glutathione in cumulus cells from women (Turathum et al., 2022), reinforcing potential impaired antioxidant mechanisms with maternal aging. In old mares, pyroglutamic acid was strongly correlated between all cell types, with only a tendency to be elevated at 24 h in cumulus cells.

Another amino acid, alanine, is thought to have a function in oocyte maturation and developmental potential (Lee et al., 2019). Lactate dehydrogenase catalyzes the interconversion of pyruvate and lactate, and oocytes utilize lactate-derived pyruvate almost exclusively to produce alanine (Dumollard et al., 2007). In this study, a reduced abundance of alanine was observed in oocytes from old when compared to young mares at 24 h of follicular maturation. Alanine can also be transferred from cumulus cells to oocytes (Su et al., 2009), but lower alanine was also observed in granulosa and cumulus cells from old when compared to young mares, demonstrating that its impaired synthesis might not be restricted to the oocyte. Although we cannot determine the mechanisms associated with altered alanine abundance in older mares’ follicles, the concentration of alanine in follicular fluid correlates positively to oocyte developmental potential in women and cows (Sinclair et al., 2008; Matoba et al., 2014), suggesting that mechanisms associated with lower alanine in old mares’ follicle cells could relate to lower oocyte developmental potential, as in the other species.

Putrescine is a polyamine derived from arginine catabolism that plays important roles in cell proliferation and differentiation and in antioxidant function (Hussain et al., 2017). The specific roles of putrescine in female reproduction are still elusive; but putrescine concentration rises in follicular cells and oocytes during LH-induced follicular maturation and is reduced in follicles of aged female rodents and women (Tao and Liu, 2013; Tao et al., 2019). Putrescine supplementation, through diet or culture medium, improved maturation rates and quality of oocytes from old female mice (Tao et al., 2019; Shi et al., 2022). Interestingly, our results show opposite trends for putrescine with mare aging, with higher abundance of the polyamine in oocytes and follicular cells from old in comparison to young mares at the 24h and 42 h maturation timepoints. Putrescine in the ovarian follicle of mares has not been a topic of previous investigation; but total polyamines are more abundant in mare follicular fluid than serum and equally abundant in early dominant, late dominant, and preovulatory follicles (Gérard et al., 2002). Whether elevated putrescine is beneficial or detrimental for the developing equine oocyte remains to be clarified. In sows, urine concentration of putrescine is negatively correlated with liter size (Chen et al., 2019), suggesting that the relationship of putrescine and fertility may not be well defined in all species. In rats, the intraperitoneal injection of putrescine limits systemic lipid metabolism, potentially by inhibiting lipases (Shelepov et al., 1990). Thus, there may be a link between elevated putrescine and triglyceride accumulation in the preovulatory follicles of old mares.

In addition to energy substrates and amino acids and their derivatives, multiple other compounds were observed to differ with equine maternal aging. Adenosine is a purine nucleoside present in follicular fluid with major role in promoting oocyte meiotic arrest (Salustri et al., 1988; Caballero et al., 2020). Oocytes gradually uptake adenosine from the follicular environment and phosphorylate it for production of ATP and regulation of cAMP levels (Salustri et al., 1988). After ovulation, the oocyte loses the support of the follicular environment, and its integrity will be largely dependent on adenosine nucleotides and substrates acquired before ovulation (Wen et al., 2010). In human ovarian follicles, adenosine follicular fluid concentration decreases with increasing follicular size, and this reduction reflects follicular maturation (Wen et al., 2010); similar findings have also been observed in mice after induction of follicular maturation (Eppig et al., 1985). In the present study, we observed higher adenosine in granulosa cells from old than young mares at 24 and 42 h, representing metaphase I and II stages of maturation, and lower adenosine in oocytes at 42 h in old than young mares. These findings could reflect ineffective reduction in follicular adenosine concentration in old mares as well as limited adenosine uptake by their oocytes, likely contributing to impaired oocyte maturation, energy production, and timing of meiotic resumption (Cheon, 2016). Age-related disturbances in follicular adenosine have not been described in other species and could represent an area for future investigations.

Phosphoric acid is a cellular source of phosphates for many essential biological processes (Kamerlin et al., 2013). Phosphoric acid concentration is maximal in developmentally competent murine oocytes as soon as they reach maturation in comparison to pre- and post-maturation oocytes (Ishigaki et al., 2019). In the present study, phosphoric acid was more abundant from old than young mare oocytes at 0 h and granulosa and cumulus cells at 24 h. The impact of elevated phosphoric acid in follicular cells and oocytes of old mares on oocyte maturation and quality has not previously been reported and is unknown. β-sitosterol is a phytosterol present in many plants including alfalfa (Shah et al., 2020). The elevated abundance of β-sitosterol in oocytes and granulosa cells from old than young mares may have come from dietary alfalfa and potentially different systemic metabolization of this phytosterol between the age groups. When fed at high levels, typically not present in alfalfa, phytosterols can affect female fertility by impairing follicular dominance and ovulation (Mostrom and Evans, 2011), but these phenomena were not observed in old mares during our experiment.

Granulosa cell mitochondria play an important role in steroidogenesis, and mitochondrial dysfunction, and damage associated with maternal aging lessens estrogen production in women (Lejri et al., 2018). Whilst no effect of advanced mare age has been reported on circulating estradiol or progesterone (Carnevale et al., 1993; Ginther et al., 2009), lower abundance of cholesterol metabolites observed in granulosa cells from old than young mares at 24h and 42 h maturation time points could indicate changes in steroidogenic pathways, as suggested by higher *STAR* transcript abundance in granulosa cells from old than young mares previously reported by our group (Sessions-Bresnahan and Carnevale, 2015). The steroidogenic acute regulatory protein (STAR) is responsible for cholesterol transport into the mitochondria for the first step of steroidogenesis.

The secondary aim of this study was to correlate metabolic activity among cell types, attempting to highlight potential mechanistic differences in which each cell contributes to oocyte development. To the best of our knowledge, correlations between metabolomic profiles of oocytes, cumulus and granulosa cells obtained from individual follicles have not been reported, although the metabolome profiles of cumulus and granulosa cells collected from the same preovulatory follicle of women have been compared (Gao et al., 2022). In the present study, we observed several metabolites with significant correlations among different cell types when obtained from the follicles of young or old mares. As we expected, relatively few were observed between oocytes and somatic follicular cells (cumulus or granulosa cells) in both age groups, likely due to differences in substrate metabolism, active metabolic pathways, and energy demands. In contrast, many correlations were observed between granulosa and cumulus cells. Granulosa cells and cumulus cells must rapidly proliferate to support the oocyte, produce steroid hormones, and induce ovulation, while the oocyte is relatively quiet prior to fertilization. The similarities between granulosa and cumulus cells are helpful for the study of follicle cell metabolism, as granulosa cells are much more numerous and easier to recover than cumulus cells during follicular aspiration procedures, and additional investigations into granulosa cell metabolic pathway activity, such as enzyme expression or metabolic flux analysis, will likely represent cumulus cells.

Energy substrates and amino acids were some of the compounds most closely coordinated among cell types, although the strength of correlations was, at times, affected by mare age. Glucose abundance was correlated between granulosa and cumulus cells, although more strongly in young than old mares. However, glucose abundance was strongly correlated between oocytes and granulosa cells for both age groups, perhaps demonstrating that this essential monosaccharide can effectively be transferred from the follicular environment to the oocyte in the mare, as observed in mice (Wang et al., 2012). A strong correlation was noted for sorbose in cumulus and granulosa cells for the young mares; in old mares, weaker but significant correlations were noted among all cell types. In young mares, myo-inositol was among the strongest correlations observed between oocytes and cumulus cells, as well as between cumulus and granulosa cells. For old mares, correlations were weaker and similar among all cell types. Free fatty acids (including octadecenoic, octadecanoic, octadecadienoic and hexadecanoic) were amongst the strongest correlated metabolites between oocytes and cumulus cells in both age groups. Strong correlations were observed between amino acids in cumulus and granulosa cells, consistent with a recent human study in which amino acids were moderately or strongly correlated between cumulus and granulosa cells (Gao et al., 2022). In mice during follicular development, the oocyte regulates amino acid uptake by cumulus and granulosa cells via paracrine factors (Eppig et al., 2005). Therefore, the strong correlations that we observed in amino acid abundance was not surprising. Notably, we observed more strong and very strong correlations for amino acids between cumulus and granulosa cells from young when compared to old mares, which may reflect a lack of appropriate communication among cell types in the old mares. In addition, glutamic acid abundance was very strongly correlated among cumulus and granulosa cells in both age groups. The close association among ovarian cell types provides challenges when studying oocyte and follicle cell function. Correlations among cell types in the present study demonstrate the likely coordination and interdependence among cells within the ovarian follicle, although even these aspects of ovarian function were affected by maternal aging and warrant further study.

In addition to the annotated metabolites, numerous unidentified metabolites were significantly affected by maternal age. Although we were unable to identify these metabolites, they suggest that additional metabolic pathways are affected by maternal aging in all 3 cell types. Future investigations into oocyte and follicle cell metabolism may use the present study to target likely affected pathways, such as the pentose-phosphate pathway, one carbon metabolism, and the citric acid cycle. Additional information regarding changes to these pathways could identify key areas of dysfunction and lead to targeted approaches to supplement oocytes for deficient compounds, *in vivo* or *in vitro*, in older animals to improve cellular function and fertility.

**FIGURE 8:**
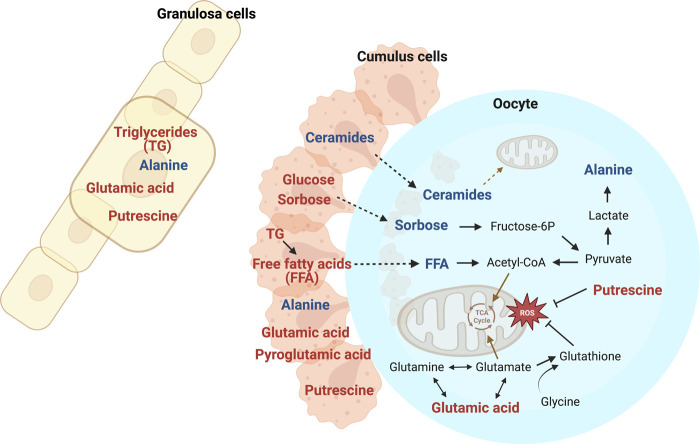
Selected metabolites affected by mare age in oocytes, cumulus cells, and granulosa cells, with metabolites elevated in old mares (red) and young mares (blue) relative to the other age group and potentially affected metabolic pathways. Transport of carbohydrates and free fatty acids (FFA) from cumulus cells to oocytes appeared impaired in old mares, potentially limiting oocyte metabolic capacity, including availability of acetyl-CoA for the TCA cycle. Relatively higher abundance of triglycerides (TG) in granulosa and cumulus cells from old mares may indicate impaired utilization of these energy reserves. Alanine is derived from lactate, and the greater abundance of alanine in all cell types for young than old mares could indicate impaired anaerobic metabolism with aging. Glutamic acid is crucial for glutamate and glutathione synthesis; higher abundance in old mares could be related to limited substrates for the TCA cycle and less antioxidant potential. Putrescine seems to act as an antioxidant, and ceramides are incorporated into mitochondrial membranes and aid in their function. Created with Biorender.com.

In summary, we used a nontargeted metabolomics approach to investigate the effects of mare maternal aging on different cell types within the ovarian follicle at distinct time points during maturation of the dominant, follicular-phase follicle. The metabolomic analyses from single oocytes and the associated cumulus and granulosa cells allowed us to correlate metabolite abundance between the different cell types. Overall, many of our age-related findings point to impaired mitochondrial metabolic function and the associated oxidative stress in oocytes and follicular cells from old mares, reinforcing previous reports in mares and other females (Bentov et al., 2011; Sessions-Bresnahan and Carnevale, 2015; Liu et al., 2017; Catandi et al., 2021; Alberico and Woods, 2022; Lu et al., 2022; Smits et al., 2023). Supporting findings include higher abundance of glutamic acid and triglycerides and lower abundance of ceramides in oocytes and somatic follicular cells from old than young mares (Figure 8). Some of our findings, especially the lower abundance of alanine in all follicular cell types from old mares, suggest limited anaerobic energy metabolism, also supported by our previous studies (Catandi et al., 2021). Results of the present study also indicate potential impaired transfer of carbohydrate and free fatty acid substrates from cumulus cells to the oocyte in old mares, likely related to disruption of the transzonal projections between cumulus cells and oocytes. The identification of these metabolic differences and related pathways may contribute to development of therapeutic interventions targeting age-induced follicular cell dysfunction. We highlighted strongly correlated metabolites among the different cell types in the follicle in both age groups, which contributes to our understanding of the integrated cellular environment, and the potential breakdown in this synergy within the follicle of the old mare.

## Data Availability

The original contributions presented in the study are included in the article/Supplementary Material, further inquiries can be directed to the corresponding author. We recently made all the metabolomics raw dataset publicly available on Figshare at this link: https://figshare.com/s/535e7d4f5b9aaf0340d9.

## Supplementary material

Supplementary Figure S1 Heatmaps of relative abundance of annotated metabolites identified in oocytes from old and young mares (red and green bars, respectively, above heatmaps) at **(A)** 0 h, **(B)** 24 h, and **(C)** 42 h of maturation. Different metabolite classes and associated compounds are labeled with different colored boxes: carbohydrates and derivatives in blue, free fatty acids in yellow, amino acids in orange, triacylglycerols in purple, diacylglycerols and monoacylglycerols in green, ceramides in brown, cholesterol esters in pink, sphingomyelins in grey, and phospholipids in red.

Supplementary Figure S2 Heatmaps of relative abundance of annotated metabolites identified in cumulus cells from old and young mares (red and green bars, respectively, above heatmaps) at **(A)** 0 h, **(B)** 24 h, and **(C)** 42 h of maturation. Different metabolite classes and associated compounds are labeled with different colored boxes: carbohydrates and derivatives in blue, free fatty acids in yellow, amino acids in orange, triacylglycerols in purple, diacylglycerols and monoacylglycerols in green, ceramides in brown, cholesterol esters in pink, sphingomyelins in grey, and phospholipids in red.

Supplementary Figure S3 Heatmaps of relative abundance of annotated metabolites identified in granulosa cells from old and young mares (red and green bars, respectively, above heatmaps) at **(A)** 0 h, **(B)** 24 h, and **(C)** 42 h of maturation. Different metabolite classes and associated compounds are labeled with different colored boxes: carbohydrates and derivatives in blue, free fatty acids in yellow, amino acids in orange, triacylglycerols in purple, diacylglycerols and monoacylglycerols in green, ceramides in brown, cholesterol esters in pink, sphingomyelins in grey, and phospholipids in red.
