# Supplementary material for: Equine maternal aging affects the metabolomic profile of oocytes and follicular cells during different maturation time points
Source: Front Cell Dev Biol. 2023 Sep 25;11:1239154. doi: 10.3389/fcell.2023.1239154 (PMC10561129; doi:10.3389/fcell.2023.1239154)
Supplement: Supplementary file 2 [file Table1.DOCX]

**Supplementary Table 1.** Relative abundance of annotated oocyte metabolites that were altered by mare age at 0h, 24h and 42h of maturation time (Time). Single oocytes analyzed from young mares (Young, n=8 for all time points) and old mares (Old; 0h, n=10; 24h, n=14; 42h, n=9). Results presented as mean ± SEM for metabolites with P-values (PV) <0.1; CF denotes correction factor for each metabolite.

| Time | Class | Metabolite | Young | Old | CF | PV |
| --- | --- | --- | --- | --- | --- | --- |
| 0h | Carbohydrates and derivatives | Pyruvic acid | 5.43 ± 0.18 | 4.77 ± 0.23 | 10^6^ | 0.038 |
|  |  | Sorbose | 9.15 ± 0.71 | 7.40 ± 0.33 | 10^5^ | 0.051 |
|  |  | Xylose | 9.08 ± 0.54 | 7.60 ± 0.42 | 10^5^ | 0.048 |
|  | Lipids | TG(52:5) | 2.61 ± 0.33 | 4.46 ± 0.81 | 10^4^ | 0.059 |
|  |  | Cer(d42:1) | 7.29 ± 1.22 | 4.82 ± 0.41 | 10^3^ | 0.089 |
|  |  | Cer(d41:1) | 3.08 ± 0.32 | 2.37 ± 0.20 | 10^3^ | 0.085 |
|  |  | Cer(d40:1) | 4.34 ± 0.57 | 3.11 ± 0.38 | 10^3^ | 0.097 |
|  |  | GlcCer(d42:1) | 3.10 ± 0.45 | 2.13 ± 0.29 | 10^3^ | 0.097 |
|  |  | DG(O-36:4) | 3.04 ± 0.38 | 1.86 ± 0.26 | 10^3^ | 0.024 |
|  |  | DG(32:1) | 1.28 ± 0.11 | 2.02 ± 0.33 | 10^4^ | 0.062 |
|  |  | DG(34:0) | 0.85 ± 0.10 | 1.60 ± 0.38 | 10^4^ | 0.087 |
|  |  | DG(40:8) | 4.08 ± 0.21 | 3.27 ± 0.33 | 10^3^ | 0.061 |
|  |  | PC(32:0) | 5.25 ± 1.22 | 2.49 ± 0.49 | 10^4^ | 0.063 |
|  |  | PE(O-36:2) | 9.66 ± 1.75 | 5.29 ± 0.51 | 10^3^ | 0.042 |
|  |  | 1-[(11Z,14Z)-11,14-Nonadecadienoyloxy]-3-[(9Z)-9-tetradecenoyloxy]-2-propanyl (11Z)-11-icosenoate | 3.79 ± 0.36 | 5.50 ± 0.68 | 10^4^ | 0.047 |
|  |  | (3beta,5xi,16alpha)-16-Hydroxy-3-{[beta-D-xylopyranosyl-(1->2)-alpha-L-arabinopyranosyl-(1->6)-2-acetamido-2-deoxy-beta-D-glucopyranosyl]oxy}olean-12-en-28-oic acid | 5.93 ± 1.29 | 2.79 ± 0.53 | 10^2^ | 0.051 |
|  | Amino acids | Glutamic acid | 0.68 ± 0.06 | 1.31 ± 0.32 | 10^6^ | 0.091 |
|  |  | Glycine (2TMS) | 7.31 ± 0.25 | 6.21 ± 0.27 | 10^7^ | 0.009 |
|  | Miscellaneous | Phosphoric acid | 2.58 ± 0.37 | 3.93 ± 0.42 | 10^7^ | 0.028 |
|  |  | Phosphoric acid monomethyl ester | 1.30 ± 0.21 | 2.09 ± 0.30 | 10^6^ | 0.047 |
|  |  | Beta-sitosterol | 0.73 ± 0.18 | 1.27 ± 0.18 | 10^4^ | 0.054 |
| 24h | Carbohydrates | Sorbose | 8.88 ± 0.44 | 7.76 ± 0.44 | 10^5^ | 0.087 |
|  | Lipids | GalCer(d42:2) | 2.37 ± 0.41 | 5.51 ± 2.09 | 10^3^ | 0.072 |
|  |  | DG(42:10) | 1.96 ± 0.34 | 1.26 ± 0.11 | 10^3^ | 0.082 |
|  |  | PC(30:0) | 3.72 ± 0.46 | 6.29 ± 1.35 | 10^3^ | 0.091 |
|  |  | [(E,2S,3R)-3-hydroxy-2-(pentadecanoylamino) octadec-4-enyl] 2-(trimethylammonio)ethyl phosphate | 0.74 ± 0.14 | 1.43 ± 0.26 | 10^3^ | 0.031 |
|  | Amino acids | Glutamic acid | 0.77 ± 0.13 | 1.26 ± 0.21 | 10^6^ | 0.066 |
|  |  | Alanine | 1.12 ± 0.07 | 0.88 ± 0.06 | 10^7^ | 0.019 |
|  |  | Glycine (3TMS) | 2.71 ± 0.07 | 2.94 ± 0.06 | 10^7^ | 0.020 |
|  | Miscellaneous | Putrescine | 0.24 ± 0.02 | 2.58 ± 0.84 | 10^7^ | 0.016 |
|  |  | 2-hydroxy-pyridine | 4.17 ± 0.32 | 5.08 ± 0.27 | 10^8^ | 0.043 |
| 42h | Lipids | Octadecanoic acid | 1.03 ± 0.15 | 0.67 ± 0.08 | 10^7^ | 0.068 |
|  |  | Octadecenoic acid | 1.33 ± 0.14 | 0.96 ± 0.09 | 10^6^ | 0.042 |
|  |  | Octadecadienoic acid | 1.83 ± 0.51 | 0.82 ± 0.12 | 10^5^ | 0.089 |
|  |  | Hexadecanoic acid | 1.46 ± 0.14 | 1.13 ± 0.07 | 10^7^ | 0.054 |
|  |  | Cer(40:1) | 4.47 ± 0.33 | 3.07 ± 0.30 | 10^3^ | 0.007 |
|  |  | PC(32:2) | 8.21 ± 0.89 | 6.17 ± 0.67 | 10^3^ | 0.088 |
|  |  | PC(P-39:1) | 6.54 ± 1.05 | 3.57 ± 1.05 | 10^4^ | 0.065 |
|  |  | 3-(Tetradecanoyloxy)-1,2-propanediyl (9Z,9'Z)bis(-9-hexadecenoate) | 1.06 ± 0.11 | 1.51 ± 0.21 | 10^4^ | 0.084 |
|  | Amino acids | Glutamic acid | 1.00 ± 0.24 | 2.04 ± 0.44 | 10^6^ | 0.061 |
|  |  | Glycine (3TMS) | 3.02 ± 0.17 | 3.55 ± 0.25 | 10^7^ | 0.097 |
|  |  | Glycine (2TMS) | 7.37 ± 0.26 | 6.33 ± 0.37 | 10^7^ | 0.039 |
|  | Miscellaneous | Putrescine | 0.24 ± 0.02 | 1.44 ± 0.61 | 10^7^ | 0.085 |
|  |  | Adenosine | 4.53 ± 0.46 | 3.00 ± 0.39 | 10^4^ | 0.023 |
|  |  | Beta-sitosterol | 0.64 ± 0.18 | 1.65 ± 0.32 | 10^4^ | 0.017 |

**Supplementary Table 2.** Relative abundance of annotated cumulus cell metabolites affected by mare age. Cumulus cells were analyzed from Young (0h, 24h, 42h n=8) and Old (0h n=8, 24h n=14, 42h n=10). Results are presented as mean ± SEM.

| Time | Class | Metabolite | Young | Old | CF | PV |
| --- | --- | --- | --- | --- | --- | --- |
| 0h | Lipids | Octadecanoic acid | 1.28 ± 0.17 | 0.91 ± 0.10 | 10^7^ | 0.092 |
|  |  | TG(52:5) | 1.85 ± 0.26 | 4.05 ± 0.74 | 10^4^ | 0.022 |
|  |  | TG(58:5) | 1.99 ± 0.24 | 3.31 ± 0.36 | 10^3^ | 0.010 |
|  |  | TG(62:14) | 1.15 ± 0.15 | 2.42 ± 0.51 | 10^3^ | 0.042 |
|  |  | Cer(d40:1) | 6.41 ± 0.66 | 4.15 ± 0.77 | 10^3^ | 0.044 |
|  |  | LPE(18:0) | 7.91 ± 1.61 | 3.49 ± 0.73 | 10^3^ | 0.032 |
|  |  | PE(O-36:2) | 7.83 ± 1.12 | 4.77 ± 0.71 | 10^3^ | 0.040 |
|  | Amino acids | Glutamic acid | 0.52 ± 0.04 | 1.43 ± 0.41 | 10^6^ | 0.060 |
|  |  | Cysteine | 1.75 ± 0.27 | 1.21 ± 0.10 | 10^5^ | 0.097 |
|  |  | Glycine (2TMS) | 6.76 ± 0.54 | 4.98 ± 0.45 | 10^7^ | 0.024 |
| 24h | Carbohydrates | Glucose | 0.33 ± 0.09 | 1.16 ± 0.35 | 10^8^ | 0.038 |
|  |  | Sorbose | 1.04 ± 0.08 | 1.77 ± 0.31 | 10^6^ | 0.036 |
|  | Lipids | TG(52:5) | 2.60 ± 0.31 | 4.93 ± 0.78 | 10^4^ | 0.012 |
|  |  | TG(56:7) | 1.82 ± 0.32 | 3.51 ± 0.63 | 10^4^ | 0.026 |
|  |  | TG(58:6) | 5.27 ± 1.07 | 9.37 ± 1.35 | 10^3^ | 0.027 |
|  |  | TG(58:8) | 2.48 ± 0.34 | 3.73 ± 0.45 | 10^4^ | 0.039 |
|  |  | TG(54:7) | 1.14 ± 0.24 | 2.29 ± 0.44 | 10^4^ | 0.033 |
|  |  | TG(52:5) | 2.01 ± 0.40 | 4.49 ± 0.81 | 10^4^ | 0.013 |
|  |  | TG(56:8) | 1.19 ± 0.35 | 2.56 ± 0.46 | 10^4^ | 0.027 |
|  |  | TG(58:5) | 3.02 ± 0.27 | 4.16 ± 0.46 | 10^3^ | 0.046 |
|  |  | TG(56:5) | 2.05 ± 0.30 | 3.11 ± 0.36 | 10^4^ | 0.036 |
|  |  | TG(54:8) | 2.41 ± 0.47 | 6.04 ± 1.24 | 10^3^ | 0.014 |
|  |  | Cer(d34:0) | 2.74 ± 0.38 | 1.75 ± 0.17 | 10^3^ | 0.038 |
|  |  | Cer(d34:1) | 3.61 ± 0.38 | 2.56 ± 0.30 | 10^4^ | 0.047 |
|  |  | CE(18:2) | 1.88 ± 0.31 | 1.01 ± 0.13 | 10^5^ | 0.029 |
|  |  | PC(30:0) | 2.01 ± 0.33 | 3.42 ± 0.50 | 10^4^ | 0.030 |
|  | Amino acids | Glutamic acid | 0.74 ± 0.09 | 1.33 ± 0.23 | 10^6^ | 0.029 |
|  |  | Glycine (2TMS) | 6.69 ± 0.32 | 4.74 ± 0.50 | 10^7^ | 0.004 |
|  | Miscellaneous | Pyroglutamic acid | 0.55 ± 0.07 | 1.35 ± 0.42 | 10^7^ | 0.084 |
|  |  | Phosphoric acid | 0.51 ± 0.10 | 1.06 ± 0.19 | 10^8^ | 0.019 |
|  |  | Phosphoric acid monomethyl ester | 3.30 ± 0.58 | 8.10 ± 0.25 | 10^6^ | 0.085 |
|  |  | Myo-inositol | 2.56 ± 0.46 | 7.02 ± 2.08 | 10^6^ | 0.055 |
| 42h | Lipids | Octadecadienoic acid | 3.81 ± 0.46 | 7.64 ± 1.79 | 10^4^ | 0.065 |
|  |  | TG(56:5) | 4.53 ± 0.73 | 5.59 ± 0.62 | 10^4^ | 0.048 |
|  |  | GlcCer(d42:1) | 3.41 ± 0.44 | 5.28 ± 0.69 | 10^3^ | 0.038 |
|  |  | DG(42:10) | 5.84 ± 0.98 | 9.09 ± 1.08 | 10^3^ | 0.041 |
|  |  | PC(O-16:0) | 2.67 ± 0.32 | 1.62 ± 0.34 | 10^5^ | 0.037 |
|  |  | PC(39:1) | 1.71 ± 0.27 | 3.07 ± 0.54 | 10^3^ | 0.041 |
|  | Amino acids | Alanine | 3.15 ± 0.35 | 2.08 ± 0.28 | 10^7^ | 0.031 |
|  | Miscellaneous | Putrescine | 0.71 ± 0.05 | 8.30 ± 3.71 | 10^6^ | 0.071 |

**Supplementary Table 3.** Relative abundance of annotated granulosa cell metabolites affected by mare age. Granulosa cells were analyzed from Young (0h n=8, 24h, 42h n=6) and Old (0h n=11, 24h n=15, 42h n=9). Results are presented as mean ± SEM.

| Time | Class | Metabolite | Young | Old | CF | PV |
| --- | --- | --- | --- | --- | --- | --- |
| 0h | Lipids | DG(36:4) | 2.68 ± 0.46 | 4.05 ± 0.33 | 10^4^ | 0.030 |
|  |  | DG(38:4) | 0.84 ± 0.13 | 1.12 ± 0.87 | 10^5^ | 0.094 |
|  |  | DG(O-36:4) | 2.34 ± 0.20 | 1.59 ± 0.27 | 10^3^ | 0.044 |
|  |  | SM(d32:1) | 3.62 ± 0.38 | 4.65 ± 0.37 | 10^3^ | 0.071 |
|  |  | PC(36:2) | 0.84 ± 0.13 | 1.12 ± 0.09 | 10^5^ | 0.094 |
|  |  | PE(38:2) | 7.11 ± 0.77 | 5.41 ± 0.44 | 10^4^ | 0.006 |
|  |  | PE(38:1) | 6.74 ± 0.23 | 5.46 ± 0.55 | 10^4^ | 0.052 |
|  |  | PC(36:4) | 1.86 ± 0.15 | 2.47 ± 0.16 | 10^5^ | 0.012 |
|  |  | PC(38:4) | 1.07 ± 0.08 | 1.39 ± 0.06 | 10^5^ | 0.008 |
|  |  | PC(36:2) | 8.54 ± 0.19 | 7.85 ± 0.27 | 10^5^ | 0.050 |
|  |  | PE(36:1) | 1.17 ± 0.03 | 0.93 ± 0.09 | 10^5^ | 0.026 |
|  |  | PC(35:5) | 4.09 ± 0.15 | 5.08 ± 0.26 | 10^4^ | 0.006 |
|  |  | PC(38:5) | 1.16 ± 0.14 | 1.70 ± 0.09 | 10^5^ | 0.058 |
|  |  | PE(42:2) | 1.32 ± 0.11 | 1.95 ± 0.24 | 10^4^ | 0.035 |
|  |  | PC(39:1) | 5.28 ± 0.65 | 9.33 ± 2.00 | 10^3^ | 0.081 |
|  |  | PC(38:7) | 1.43 ± 0.16 | 2.41 ± 0.20 | 10^4^ | 0.001 |
|  |  | PC(28:0) | 3.90 ± 0.88 | 8.53 ± 2.19 | 10^3^ | 0.074 |
|  |  | [(E,2S,3R)-3-hydroxy-2-(pentadecanoylamino) octadec-4-enyl] 2-(trimethylammonio) ethyl phosphate | 4.60 ± 0.40 | 3.53 ± 0.27 | 10^3^ | 0.044 |
|  |  | (3beta,5xi,16alpha)-16-Hydroxy-3-{[beta-D-xylopyranosyl-(1->2)-  alpha-L-arabinopyranosyl-(1->6)-2-acetamido-2-deoxy-beta-D-glucopyranosyl]oxy}  olean-12-en-28-oic acid | 1.84 ± 0.34 | 4.00 ± 0.73 | 10^3^ | 0.019 |
|  |  | 2,2,4,4,6,6-Hexamethyl-  1,3,5-trithiane | 1.62 ± 0.42 | 3.37 ± 0.59 | 10^3^ | 0.029 |
|  |  | (5,5-Dimethyl-2-oxotetrahydro-3-furanyl) methyl methanesulfonate | 2.14 ± 0.71 | 4.64 ± 0.90 | 10^3^ | 0.045 |
|  | Amino acids | Glycine (3TMS) | 2.35 ± 0.27 | 3.02 ± 0.15 | 10^7^ | 0.054 |
|  | Miscellaneous | Raoline | 8.20 ± 0.69 | 6.10 ± 0.64 | 10^4^ | 0.041 |
| 24h | Carbohydrate derivatives | Pyruvic acid | 6.57 ± 0.50 | 4.92 ± 0.76 | 10^6^ | 0.088 |
|  | Lipids | Octadecenoic acid | 4.22 ± 0.77 | 6.67 ± 0.92 | 10^6^ | 0.057 |
|  |  | Linoleic acid | 0.58 ± 0.13 | 1.02 ± 0.20 | 10^3^ | 0.086 |
|  |  | TG(52:5) | 3.22 ± 0.35 | 4.73 ± 0.66 | 10^4^ | 0.060 |
|  |  | TG(56:7) | 4.09 ± 0.11 | 5.57 ± 0.49 | 10^4^ | 0.075 |
|  |  | TG(54:7) | 1.23 ± 0.14 | 1.93 ± 0.22 | 10^4^ | 0.016 |
|  |  | TG(58:5) | 0.69 ± 0.12 | 1.09 ± 0.16 | 10^4^ | 0.068 |
|  |  | TG(48:1) | 0.85 ± 0.04 | 1.27 ± 0.16 | 10^4^ | 0.028 |
|  |  | TG(56:5) | 4.00 ± 0.40 | 5.78 ± 0.54 | 10^4^ | 0.017 |
|  |  | TG(49:1) | 2.37 ± 0.22 | 3.66 ± 0.51 | 10^3^ | 0.035 |
|  |  | Cer(42:2) | 1.99 ± 0.44 | 3.25 ± 0.44 | 10^4^ | 0.064 |
|  |  | Cer(34:1) | 1.89 ± 0.46 | 0.95 ± 0.12 | 10^4^ | 0.098 |
|  |  | Cer(40:1) | 0.97 ± 0.16 | 1.70 ± 0.29 | 10^4^ | 0.043 |
|  |  | Cer(d42:2) | 0.56 ± 0.24 | 2.01 ± 0.57 | 10^3^ | 0.034 |
|  |  | GalCer(d42:2) | 5.94 ± 1.31 | 9.44 ± 1.06 | 10^3^ | 0.060 |
|  |  | GlcCer(d42:1) | 5.26 ± 1.09 | 9.15 ± 1.12 | 10^3^ | 0.026 |
|  |  | DG(38:4) | 1.91 ± 0.33 | 4.50 ± 1.04 | 10^4^ | 0.033 |
|  |  | DG(36:4) | 0.58 ± 0.06 | 1.52 ± 0.33 | 10^4^ | 0.015 |
|  |  | DG(32:1) | 2.74 ± 0.52 | 3.96 ± 0.33 | 10^3^ | 0.081 |
|  |  | DG(40:8) | 1.71 ± 0.20 | 2.64 ± 0.37 | 10^3^ | 0.042 |
|  |  | DG(20:7) | 0.55 ± 0.13 | 1.59 ± 0.51 | 10^3^ | 0.067 |
|  |  | DG(42:10) | 1.55 ± 0.21 | 2.25 ± 0.30 | 10^3^ | 0.073 |
|  |  | 5a-Cholestanol | 2.74 ± 0.58 | 1.42 ± 0.21 | 10^3^ | 0.077 |
|  |  | Cholestenone | 2.08 ± 0.17 | 1.19 ± 0.11 | 10^4^ | 0.002 |
|  |  | SM(24:0) | 1.99 ± 0.33 | 3.21 ± 0.51 | 10^4^ | 0.060 |
|  |  | PC(34:0) | 2.79 ± 0.12 | 2.39 ± 0.12 | 10^5^ | 0.031 |
|  |  | PC(36:4) | 1.85 ± 0.26 | 2.52 ± 0.27 | 10^5^ | 0.091 |
|  |  | PC(36:1) | 3.73 ± 0.13 | 4.07 ± 0.13 | 10^5^ | 0.064 |
|  |  | PE(42:0) | 0.83 ± 0.15 | 1.22 ± 0.07 | 10^4^ | 0.048 |
|  |  | PC(36:5) | 1.18 ± 0.21 | 0.61 ± 0.11 | 10^5^ | 0.047 |
|  |  | PC(35:4) | 2.34 ± 0.43 | 4.85 ± 0.53 | 10^3^ | 0.002 |
|  |  | LPC(18:3) | 0.52 ± 0.16 | 1.25 ± 0.36 | 10^4^ | 0.087 |
|  |  | PE(P-38:4) | 4.40 ± 0.72 | 7.15 ± 1.06 | 10^4^ | 0.047 |
|  |  | PC(36:4) | 1.36 ± 0.25 | 0.80 ± 0.1 | 10^5^ | 0.085 |
|  |  | LPE(18:0) | 1.48 ± 0.20 | 2.88 ± 0.64 | 10^4^ | 0.056 |
|  |  | PE(42:2) | 1.53 ± 0.16 | 1.92 ± 0.13 | 10^4^ | 0.090 |
|  |  | PC(O-36:3) | 1.55 ± 0.15 | 1.12 ± 0.13 | 10^4^ | 0.055 |
|  |  | LPC(16:1) | 4.42 ± 0.92 | 2.27 ± 0.40 | 10^3^ | 0.070 |
|  |  | 1-[5-(1-Hydroxytridecyl) tetrahydro-2-furanyl]-13-(5-methyl-2-oxo-2,5-dihydro-3-furanyl) tridecyl palmitate | 0.76 ± 0.03 | 1.07 ± 0.12 | 10^4^ | 0.028 |
|  |  | 2-(4-methylthiazol-5-yl) ethyl acetate | 2.50 ± 0.48 | 1.39 ± 0.19 | 10^4^ | 0.069 |
|  |  | N-(2-stearamidoethyl) stearamide | 1.25 ± 0.25 | 0.67 ± 0.09 | 10^4^ | 0.072 |
|  |  | (2R)-2-(Palmitoyloxy)-3-(phosphonooxy)propyl heptadecanoate | 1.72 ± 0.27 | 3.51 ± 0.53 | 10^3^ | 0.008 |
|  | Amino acids | Glutamic acid | 0.81 ± 0.33 | 1.90 ± 0.33 | 10^6^ | 0.036 |
|  |  | Cysteine | 0.28 ± 0.11 | 1.08 ± 0.25 | 10^6^ | 0.010 |
|  |  | Alanine | 1.24 ± 0.33 | 0.55 ± 0.44 | 10^7^ | 0.095 |
|  |  | Glycyl-Tyrosine | 5.42 ± 0.45 | 3.78 ± 0.40 | 10^5^ | 0.017 |
|  |  | Glycine (3TMS) | 2.85 ± 0.38 | 4.53 ± 0.44 | 10^7^ | 0.010 |
|  | Miscellaneous | Phosphoric acid | 1.62 ± 0.45 | 3.44 ± 0.47 | 10^8^ | 0.014 |
|  |  | Phosphoric acid monomethyl ester | 2.73 ± 0.85 | 6.22 ± 0.67 | 10^7^ | 0.008 |
|  |  | Myo-inositol | 4.03 ± 1.42 | 7.75 ± 1.15 | 10^6^ | 0.064 |
|  |  | Putrescine | 0.42 ± 0.32 | 1.61 ± 0.55 | 10^7^ | 0.075 |
|  |  | Adenosine | 0.72 ± 0.23 | 1.34 ± 0.24 | 10^6^ | 0.084 |
|  |  | Lipoyl-GMP | 1.06 ± 0.17 | 0.55 ± 0.08 | 10^4^ | 0.085 |
|  |  | Myo-inositol-2-phosphate | 0.78 ± 0.11 | 1.44 ± 0.19 | 10^6^ | 0.008 |
|  |  | dihydroxy- tetranor vitamin D3 | 2.73 ± 0.55 | 5.99 ± 0.86 | 10^3^ | 0.005 |
|  |  | 9-(4-Hydroxybutyl)-N2-Phenylguanine | 5.97 ± 1.60 | 2.65 ± 0.28 | 10^3^ | 0.092 |
| 42h | Lipids | Cer(42:2) | 1.10 ± 0.28 | 2.10 ± 0.48 | 10^3^ | 0.097 |
|  |  | GalCer(42:2) | 0.55 ± 0.07 | 1.19 ± 0.25 | 10^4^ | 0.038 |
|  |  | CE(18:2) | 3.91 ± 0.95 | 1.72 ± 0.36 | 10^4^ | 0.064 |
|  |  | DG(36:2) | 1.60 ± 0.23 | 1.09 ± 0.15 | 10^4^ | 0.095 |
|  |  | SM(24:0) | 3.49 ± 0.60 | 5.46 ± 0.90 | 10^4^ | 0.091 |
|  |  | PE(36:1) | 6.23 ± 0.35 | 5.16 ± 0.26 | 10^4^ | 0.029 |
|  |  | PE(36:3) | 4.67 ± 0.27 | 3.25 ± 0.35 | 10^4^ | 0.007 |
|  |  | PE(P-36:2) | 2.36 ± 0.19 | 1.78 ± 0.26 | 10^4^ | 0.096 |
|  | Amino acids | Glycine (3TMS) | 1.15 ± 0.14 | 1.59 ± 0.20 | 10^8^ | 0.093 |
|  | Miscellaneous | Beta-sitosterol | 2.66 ± 0.80 | 4.73 ± 0.78 | 10^3^ | 0.090 |
|  |  | Adenosine | 1.67 ± 0.50 | 3.50 ± 0.76 | 10^6^ | 0.093 |
