# Supplementary material for: Equine maternal aging affects the metabolomic profile of oocytes and follicular cells during different maturation time points
Source: Front Cell Dev Biol. 2023 Sep 25;11:1239154. doi: 10.3389/fcell.2023.1239154 (PMC10561129; doi:10.3389/fcell.2023.1239154)
Supplement: Supplementary file 5 [file Table2.DOCX]

**Supplementary Table 4.** Annotated metabolites with correlations (P<0.1) between different follicular cell types collected from young mares.

| Follicular cell types | Class | Metabolite | Correlation coefficient | P-value |
| --- | --- | --- | --- | --- |
| Oocyte and Cumulus cells | Lipids | Octadecenoic acid | 0.66 | 0.0005 |
| (n=24) |  | Octadecanoic acid | 0.62 | 0.001 |
|  |  | Hexadecanoic acid | 0.57 | 0.004 |
|  |  | TG(62:14) | 0.60 | 0.002 |
|  |  | TG(58:9) | 0.46 | 0.023 |
|  |  | Cer(42:2) | 0.37 | 0.073 |
|  |  | GalCer(d42:2) | 0.41 | 0.045 |
|  |  | CE(22:5) | 0.42 | 0.042 |
|  |  | CE(20:2) | 0.52 | 0.009 |
|  |  | DG(34:1) | 0.52 | 0.009 |
|  |  | DG(36:4) | 0.36 | 0.088 |
|  |  | SM(24:0) | 0.36 | 0.082 |
|  |  | LPE(18:0) | 0.55 | 0.006 |
|  |  | PE(36:3) | -0.37 | 0.074 |
|  |  | (2R)-2-[(9-oxononanoyl)oxy]-3-(palmitoyloxy)propyl 2-(trimethylammonio)ethyl phosphate | 0.45 | 0.029 |
|  |  | (2R)-2-[(8-carboxyoctanoyl)oxy]-3-(palmitoyloxy)propyl 2-(trimethylammonio)ethyl phosphate | 0.39 | 0.062 |
|  |  | 4-hydroxy-3-[(1E)-5-oxo-1,7-diphenyl-1-hepten-3-yl]-6-[(E)-2-phenylvinyl]-2H-pyran-2-one | 0.53 | 0.007 |
|  |  | 17alpha-20,29,30-trinorlup-13(18)-ene | 0.52 | 0.008 |
|  | Amino acids | Glycyl-tyrosine | 0.59 | 0.002 |
|  | Miscellaneous | Myo-inositol-2-phosphate | 0.60 | 0.002 |
|  |  | Pyridoxamine | 0.46 | 0.022 |
|  |  | Phosphoric acid | 0.42 | 0.043 |
|  |  | Lipoyl-GMP | 0.37 | 0.079 |
| Oocyte and Granulosa cells | Carbohydrates | Glucose | 0.68 | 0.001 |
| (n=21) |  | Sorbose | 0.50 | 0.028 |
|  | Lipids | Octadecanoic acid | 0.54 | 0.018 |
|  |  | Hexadecanoic acid | 0.48 | 0.039 |
|  |  | TG(58:6) | 0.46 | 0.037 |
|  |  | TG(56:7) | 0.38 | 0.093 |
|  |  | Cer(d41:1) | 0.40 | 0.074 |
|  |  | CE(20:2) | 0.70 | 0.0005 |
|  |  | DG(36:4) | 0.46 | 0.037 |
|  |  | DG(40:8) | 0.41 | 0.061 |
|  |  | DG(34:0) | 0.40 | 0.073 |
|  |  | PC(16:0) | 0.57 | 0.006 |
|  |  | PC(28:0) | 0.56 | 0.009 |
|  |  | PC(34:1) | 0.40 | 0.069 |
|  |  | LPE(18:1) | 0.49 | 0.024 |
|  |  | LPC(18:0) | 0.48 | 0.028 |
|  |  | LPC(16:0) | 0.45 | 0.042 |
|  |  | LPC(18:3) | 0.41 | 0.066 |
|  |  | 17alpha-20,29,30-trinorlup-13(18)-ene | 0.63 | 0.002 |
|  |  | [(E,2S,3R)-3-hydroxy-2-(pentadecanoylamino)octadec-4-enyl] 2-(trimethylammonio)ethyl phosphate | 0.43 | 0.053 |
|  |  | 4-hydroxy-3-[(1E)-5-oxo-1,7-diphenyl-1-hepten-3-yl]-6-[(E)-2-phenylvinyl]-2H-pyran-2-one | 0.43 | 0.051 |
|  | Amino acids | Glycyl-tyrosine | 0.61 | 0.003 |
|  | Miscellaneous | Lipoyl-GMP | 0.59 | 0.005 |
| Cumulus and Granulosa cells | Carbohydrates | Sorbose | 0.96 | 0.00001 |
| (n=20) |  | Glucose | 0.80 | 0.00001 |
|  | Lipids | Octadecanoic acid | 0.92 | 0.00001 |
|  |  | Hexadecanoic acid | 0.79 | 0.0001 |
|  |  | Octadecadienoic acid | 0.48 | 0.037 |
|  |  | Octadecenoic acid | 0.47 | 0.041 |
|  |  | Linoleic acid | 0.45 | 0.043 |
|  |  | TG(56:5) | 0.81 | 0.00001 |
|  |  | TG(58:6) | 0.71 | 0.0003 |
|  |  | TG(56:7) | 0.68 | 0.0007 |
|  |  | TG(58:5) | 0.62 | 0.003 |
|  |  | TG(56:4) | 0.62 | 0.003 |
|  |  | TG(62:14) | 0.57 | 0.007 |
|  |  | TG(58:9) | 0.55 | 0.010 |
|  |  | TG(56:8) | 0.54 | 0.012 |
|  |  | Cer(d42:2) | 0.47 | 0.032 |
|  |  | Cer(d34:1) | 0.45 | 0.039 |
|  |  | CE(20:2) | 0.87 | 0.00001 |
|  |  | CE(22:5) | 0.80 | 0.00001 |
|  |  | DG(32:0) | 0.65 | 0.001 |
|  |  | DG(42:10) | 0.60 | 0.004 |
|  |  | DG(34:0) | -0.37 | 0.095 |
|  |  | DG(36:2) | -0.48 | 0.027 |
|  |  | MG(18:3) | 0.40 | 0.070 |
|  |  | SM(d34:1) | 0.62 | 0.002 |
|  |  | SM(d32:1) | 0.60 | 0.004 |
|  |  | LPC(20:4) | 0.65 | 0.001 |
|  |  | PC(38:5) | 0.60 | 0.004 |
|  |  | PC(36:4) | 0.56 | 0.008 |
|  |  | PC(30:1) | 0.54 | 0.011 |
|  |  | PC(39:1) | 0.46 | 0.036 |
|  |  | PE(42:2) | 0.44 | 0.044 |
|  |  | PC(38:4) | 0.40 | 0.074 |
|  |  | PE(42:0) | 0.38 | 0.085 |
|  |  | PC(35:3) | -0.39 | 0.078 |
|  |  | 4-hydroxy-3-[(1E)-5-oxo-1,7-diphenyl-1-hepten-3-yl]-6-[(E)-2-phenylvinyl]-2H-pyran-2-one | 0.72 | 0.0003 |
|  |  | (2R)-2-(palmitoyloxy)-3-(phosphonooxy)propyl heptadecanoate | 0.69 | 0.0005 |
|  |  | Glycerophosphocholine | 0.61 | 0.003 |
|  | Amino acids | Glutamic acid | 0.98 | 0.00001 |
|  |  | Pyroglutamic acid | 0.98 | 0.00001 |
|  |  | Threonine | 0.95 | 0.00001 |
|  |  | Alanine | 0.88 | 0.00001 |
|  |  | Glycine | 0.88 | 0.00001 |
|  |  | Serine | 0.80 | 0.00001 |
|  |  | Cysteine | 0.71 | 0.0006 |
|  |  | Glycyl-tyrosine | 0.56 | 0.008 |
|  | Miscellaneous | Adenosine | 0.84 | 0.00001 |
|  |  | Myo-inositol | 0.79 | 0.0001 |
|  |  | 2-hydroxy-pyridine | 0.62 | 0.005 |
|  |  | Lipoyl-GMP | 0.50 | 0.020 |
|  |  | Pyridoxamine | 0.46 | 0.047 |
|  |  | Putrescine | 0.45 | 0.055 |

**Supplementary Table 5.** Annotated metabolites with correlations (P<0.1) between different follicular cell types collected from old mares.

| Follicular cell types | Class | Metabolite | Correlation coefficient | P value |
| --- | --- | --- | --- | --- |
| Oocyte and Cumulus cells | Carbohydrates | Sorbose | 0.71 | 0.00001 |
| (n=30) |  | Glucose | 0.56 | 0.001 |
|  | Lipids | Octadecadienoic acid | 0.73 | 0.00001 |
|  |  | Hexadecanoic acid | 0.70 | 0.00001 |
|  |  | Octadecanoic acid | 0.69 | 0.00001 |
|  |  | Octadecenoic acid | 0.69 | 0.00001 |
|  |  | TG(58:9) | 0.33 | 0.082 |
|  |  | TG(54:8) | -0.33 | 0.082 |
|  |  | GalCer(d42:2) | 0.34 | 0.073 |
|  |  | DG(40:8) | 0.71 | 0.00001 |
|  |  | DG(42:10) | 0.60 | 0.0006 |
|  |  | DG(34:1) | 0.50 | 0.005 |
|  |  | DG(34:3) | 0.47 | 0.010 |
|  |  | DG(36:4) | 0.46 | 0.012 |
|  |  | DG(20:7) | 0.44 | 0.017 |
|  |  | DG(34:1) | 0.42 | 0.022 |
|  |  | DG(34:2) | 0.32 | 0.093 |
|  |  | DG(36:3) | 0.32 | 0.095 |
|  |  | 17alpha-20,29,30-trinorlup-13(18)-ene | 0.48 | 0.008 |
|  |  | (2R)-2-(palmitoyloxy)-3-(phosphonooxy)propyl heptadecanoate | 0.39 | 0.037 |
|  | Amino acids | Pyroglutamic acid | 0.70 | 0.00001 |
|  |  | Threonine | 0.69 | 0.00001 |
|  |  | Glycine | 0.63 | 0.0002 |
|  |  | Alanine | 0.57 | 0.001 |
|  |  | Glutamic acid | 0.51 | 0.004 |
|  |  | Glycyl-tyrosine | 0.49 | 0.007 |
|  | Miscellaneous | Adenosine | 0.58 | 0.0009 |
|  |  | Pyridoxamine | 0.56 | 0.001 |
|  |  | Myo-inositol | 0.53 | 0.003 |
|  |  | Putrescine | 0.40 | 0.030 |
|  |  | Cysteinyl-proline | 0.38 | 0.042 |
|  |  | Myo-inositol-2-phosphate | 0.34 | 0.066 |
| Oocyte and Granulosa cells | Carbohydrates and derivatives | Sorbose | 0.76 | 0.00001 |
| (n=33) |  | Glucose | 0.71 | 0.00001 |
|  |  | Pyruvic acid | 0.31 | 0.081 |
|  | Lipids | Octadecanoic acid | 0.64 | 0.0001 |
|  |  | Hexadecanoic acid | 0.52 | 0.002 |
|  |  | Octadecenoic acid | 0.39 | 0.024 |
|  |  | TG(49:1) | 0.38 | 0.038 |
|  |  | TG(49:2) | 0.36 | 0.050 |
|  |  | TG(58:5) | -0.32 | 0.085 |
|  |  | Cer(d42:2) | 0.42 | 0.021 |
|  |  | Cer(d34:0) | 0.34 | 0.068 |
|  |  | DG(40:8) | 0.46 | 0.010 |
|  |  | DG(42:10) | 0.41 | 0.023 |
|  |  | MG(18:3) | 0.38 | 0.038 |
|  |  | LPC(18:1) | 0.54 | 0.002 |
|  |  | LPC(14:0) | 0.48 | 0.008 |
|  |  | LPC(16:1) | 0.45 | 0.013 |
|  |  | LPC(18:2) | 0.36 | 0.048 |
|  |  | PC(O-16:0) | 0.33 | 0.078 |
|  |  | PE(38:4) | -0.35 | 0.057 |
|  |  | PC(O-30:0) | -0.36 | 0.047 |
|  |  | PE(P-38:4) | -0.38 | 0.037 |
|  |  | PE(36:1) | -0.43 | 0.018 |
|  |  | PC(35:5) | -0.50 | 0.005 |
|  |  | PC(38:4) | -0.53 | 0.003 |
|  |  | ethyl 2-[(3S,4R,6R)-6-butyl-4,6-diethyldioxan-3-yl]acetate | 0.58 | 0.0008 |
|  |  | 4-hydroxy-3-[(1E)-5-oxo-1,7-diphenyl-1-hepten-3-yl]-6-[(E)-2-phenylvinyl]-2H-pyran-2-one | 0.45 | 0.012 |
|  |  | (2R)-2-(palmitoyloxy)-3-(phosphonooxy)propyl heptadecanoate | 0.33 | 0.074 |
|  |  | 1-[5-(1-hydroxytridecyl)tetrahydro-2-furanyl]-13-(5-methyl-2-oxo-2,5-dihydro-3-furanyl)tridecyl palmitate | 0.32 | 0.082 |
|  |  | 3-(13,14-dihydroxytriacontyl)-5-methyl-2(5H)-furanone | -0.32 | 0.086 |
|  | Amino acids | Threonine | 0.78 | 0.00001 |
|  |  | Pyroglutamic acid | 0.73 | 0.00001 |
|  |  | Alanine | 0.60 | 0.0002 |
|  |  | Glycine | 0.55 | 0.0008 |
|  |  | Glutamic acid | 0.45 | 0.009 |
|  |  | Glycyl-tyrosine | 0.43 | 0.017 |
|  |  | Serine | 0.37 | 0.036 |
|  |  | Cysteine | -0.37 | 0.036 |
|  | Miscellaneous | Myo-inositol | 0.53 | 0.002 |
|  |  | Myo-inositol-2-phosphate | 0.42 | 0.014 |
|  |  | Pyridoxamine | 0.42 | 0.015 |
|  |  | Adenosine | -0.43 | 0.013 |
| Cumulus and Granulosa cells | Carbohydrates and derivatives | Sorbose | 0.78 | 0.00001 |
| (n=30) |  | Pyruvic acid | 0.67 | 0.00001 |
|  |  | Glucose | 0.49 | 0.005 |
|  | Lipids | Octadecadienoic acid | 0.56 | 0.001 |
|  |  | Hexadecanoic acid | 0.48 | 0.007 |
|  |  | Octadecanoic acid | 0.46 | 0.010 |
|  |  | Octadecenoic acid | 0.40 | 0.028 |
|  |  | TG(58:9) | 0.61 | 0.0006 |
|  |  | TG(62:14) | 0.55 | 0.002 |
|  |  | TG(56:5) | 0.41 | 0.029 |
|  |  | TG(56:7) | 0.41 | 0.030 |
|  |  | TG(58:6) | 0.34 | 0.072 |
|  |  | TG(52:5) | 0.32 | 0.093 |
|  |  | GlcCer(d34:1) | 0.52 | 0.004 |
|  |  | GalCer(d42:2) | 0.47 | 0.011 |
|  |  | Cer(d42:2) | 0.34 | 0.076 |
|  |  | GlcCer(d42:1) | 0.32 | 0.095 |
|  |  | Cer(d34:0) | -0.41 | 0.032 |
|  |  | CE(20:2) | 0.78 | 0.00001 |
|  |  | CE(18:2) | 0.66 | 0.0001 |
|  |  | CE(22:5) | 0.49 | 0.008 |
|  |  | DG(40:8) | 0.75 | 0.00001 |
|  |  | DG(20:7) | 0.64 | 0.0003 |
|  |  | DG(42:10) | 0.53 | 0.004 |
|  |  | DG(32:0) | 0.48 | 0.010 |
|  |  | DG(32:1) | 0.45 | 0.017 |
|  |  | DG(34:0) | 0.33 | 0.087 |
|  |  | SM(d34:1) | 0.61 | 0.0005 |
|  |  | SM(24:0) | 0.33 | 0.089 |
|  |  | PC(28:0) | 0.79 | 0.00001 |
|  |  | PC(39:1) | 0.77 | 0.00001 |
|  |  | PE(42:0) | 0.70 | 0.00001 |
|  |  | LPC(20:4) | 0.66 | 0.0001 |
|  |  | PC(35:4) | 0.65 | 0.0002 |
|  |  | PC(P-39:1) | 0.64 | 0.0003 |
|  |  | PC(36:4) | 0.63 | 0.0004 |
|  |  | PE(42:2) | 0.59 | 0.001 |
|  |  | LPC(14:0) | 0.58 | 0.001 |
|  |  | PC(30:0) | 0.48 | 0.009 |
|  |  | LPC(16:1) | 0.44 | 0.018 |
|  |  | PC(36:5) | 0.44 | 0.019 |
|  |  | PC(37:5) | 0.44 | 0.020 |
|  |  | PC(34:1) | 0.42 | 0.027 |
|  |  | PC(30:1) | 0.39 | 0.042 |
|  |  | LPC(18:1) | 0.35 | 0.065 |
|  |  | PC(O-15:0) | 0.33 | 0.081 |
|  |  | PC(32:2) | 0.32 | 0.092 |
|  |  | LPE(18:0) | -0.33 | 0.083 |
|  |  | (2R)-2-(palmitoyloxy)-3-(phosphonooxy)propyl heptadecanoate | 0.61 | 0.0005 |
|  |  | [(E,2S,3R)-3-hydroxy-2-(pentadecanoylamino)octadec-4-enyl] 2-(trimethylammonio)ethyl phosphate | 0.55 | 0.002 |
|  |  | 4-hydroxy-3-[(1E)-5-oxo-1,7-diphenyl-1-hepten-3-yl]-6-[(E)-2-phenylvinyl]-2H-pyran-2-one | 0.53 | 0.004 |
|  |  | 9-(4-hydroxybutyl)-N2-phenylguanine | 0.50 | 0.007 |
|  |  | N-(2-stearamidoethyl)stearamide | 0.41 | 0.029 |
|  |  | 3-(tetradecanoyloxy)-1,2-propanediyl (9Z,9'Z)bis(-9-hexadecenoate) | 0.38 | 0.046 |
|  |  | 2,2,4,4,6,6-hexamethyl-1,3,5-trithiane | 0.34 | 0.072 |
|  | Amino acids | Glutamic acid | 0.92 | 0.00001 |
|  |  | Pyroglutamic acid | 0.89 | 0.00001 |
|  |  | Threonine | 0.86 | 0.00001 |
|  |  | Glycine | 0.85 | 0.00001 |
|  |  | Glycyl-tyrosine | 0.77 | 0.00001 |
|  |  | Alanine | 0.76 | 0.00001 |
|  |  | Serine | 0.48 | 0.007 |
|  |  | Cysteine | 0.47 | 0.009 |
|  | Miscellaneous | Adenosine | 0.75 | 0.00001 |
|  |  | Cysteinyl-proline | 0.62 | 0.0002 |
|  |  | Myo-inositol | 0.55 | 0.002 |
|  |  | Pyridoxamine | 0.47 | 0.008 |
|  |  | Lipoyl-GMP | 0.47 | 0.011 |
|  |  | Phosphoric acid monomethyl ester | 0.37 | 0.047 |
|  |  | Myo-inositol-2-phosphate | 0.35 | 0.060 |
